# Increased retinoic acid signaling decreases lung metastasis in salivary adenoid cystic carcinoma by inhibiting the noncanonical Notch1 pathway

**DOI:** 10.1038/s12276-023-00957-7

**Published:** 2023-03-06

**Authors:** Meng-jiao Zhou, Jia-jie Yang, Ting-yao Ma, Ge-xuan Feng, Xue-lian Wang, Li-Yong Wang, Yu-ze Ge, Ran Gao, Hong-liang Liu, Lin Shan, Lu Kong, Xiao-hong Chen

**Affiliations:** 1 Department of Otolaryngology-Head and Neck Surgery, Beijing Tongren Hospital, Capital Medical University, Beijing, 100730 China; 2 NHC Key Laboratory of Human Disease Comparative Medicine, Beijing Engineering Research Center for Experimental Animal Models of Human Critical Diseases, Institute of Laboratory Animal Sciences, Chinese Academy of Medical Sciences (CAMS) and Comparative Medicine Center, Peking Union Medical College (PUMC), Beijing, 100021 China; 3 Department of Biochemistry and Molecular Biology, Capital Medical University, Beijing, 100069 China; 4 The Central Laboratory for Molecular Biology, Capital Medical University, Beijing, 100069 China; 5 SHANDONG Longfine PHARMACEUTICAL CO., LTD, Shandong, 272622 China

**Keywords:** Metastasis, Head and neck cancer, Cancer therapy

## Abstract

*MYB-NFIB* fusion and *NOTCH1* mutation are common hallmark genetic events in salivary gland adenoid cystic carcinoma (SACC). However, abnormal expression of MYB and NOTCH1 is also observed in patients without *MYB-NFIB* fusion and *NOTCH1* mutation. Here, we explore in-depth the molecular mechanisms of lung metastasis through single-cell RNA sequencing (scRNA-seq) and exome target capture sequencing in two SACC patients without *MYB-NFIB* fusion and *NOTCH1* mutation. Twenty-five types of cells in primary and metastatic tissues were identified via Seurat clustering and categorized into four main stages ranging from near-normal to cancer-based on the abundance of each cell cluster in normal tissue. In this context, we identified the Notch signaling pathway enrichment in almost all cancer cells; RNA velocity, trajectory, and sub-clustering analyses were performed to deeply investigate cancer progenitor-like cell clusters in primary tumor-associated lung metastases, and signature genes of progenitor-like cells were enriched in the “MYC_TARGETS_V2” gene set. In vitro, we detected the NICD1-MYB-MYC complex by co-immunoprecipitation (Co-IP) and incidentally identified retinoic acid (RA) as an endogenous antagonist of genes in the “MYC_TARGETS_V2” gene set. Following this, we confirmed that all-trans retinoic acid (ATRA) suppresses the lung metastasis of SACC by correcting erroneous cell differentiation mainly caused by aberrant NOTCH1 or MYB expression. Bioinformatic, RNA-seq, and immunohistochemical (IHC) analyses of primary tissues and metastatic lung tissues from patients with SACC suggested that RA system insufficiency partially promotes lung metastasis. These findings imply the value of the RA system in diagnosis and treatment.

## Cancer: vitamin A derivative prevents salivary gland tumors spreading

Treatment of salivary gland tumors with a metabolite of vitamin A could help prevent rare cancer from spreading to the lungs. A team led by Xiao-hong Chen and Lu Kong from Capital Medical University in Beijing, China, performed extensive molecular profiling on primary and metastatic tumor cells taken from two patients with advanced salivary gland cancer. Even though these patients’ cancers did not harbor either of two common mutations that are hallmarks of the disease, both of which drive tumor proliferation and spread, the cells did show signs of altered gene expression and faulty molecular signaling consistent with these same mutational profiles. The abnormal signaling could be overcome by treatment with retinoic acid, a derivative of vitamin A that, when given to mouse models of cancer, reduced the development of lung metastases.

## Introduction

Salivary gland adenoid cystic carcinoma (SACC) has a poor clinical prognosis and a high risk of distant metastasis, most commonly to the lung^1–3^, and the 3-year lung metastasis rate can reach 24.9%^1^. The ten-year overall survival rate of patients with aggressive SACC ranges from approximately 29 to 37%^3,4^, and the median survival time of patients with lung metastatic SACC is only 20–32 months^5^. To date, there are no available treatments for metastatic SACC^6^, and in-depth exploration of the underlying mechanisms is a priority for this field.

Aberrant overexpression of MYB, NOTCH1 intracellular structural domain (NICD1), and MYC are common molecular events in SACC; MYC and NOTCH1 overexpression are mainly caused by genetic abnormalities^7–12^. However, approximately half of the cases show MYB activation in the absence of *MYB* fusion^13^, and the related mechanism remains unclear. All-trans retinoic acid (ATRA), an efficient inhibitor of MYB, induces the elevation of retinoic acid receptor α (RARα) and subsequently binds the *MYB* enhancer locus to repress *MYB* transcription or disrupt the *MYB* positive feedback loop^14^; it was recently considered to be a promising drug for aggressive SACC therapy^14,15^. Controversially, MYB fusion and upregulation are not associated with the overall survival rate^16^, whilst activating mutations in *NOTCH1* and the overexpression of *MYC* or *MYC* targets have been associated with worse outcomes and decreased overall survival in patients with SACC^11,17,18^. Thus, *NOTCH1* in SACC has been considered an important oncogene^11,19^. However, it is unclear why Notch signaling inhibitors have not achieved clinical benefit^20^. NOTCH1 mutations are present in approximately 20% of ACC patients, while the immunohistochemical positivity rate is as high as 85% (149/175 patients)^11,21^. Therefore, *NOTCH1* activation predominantly occurs in the absence of mutation.

Notch signals determine cell fate in the contexts of differentiation, proliferation, and survival and are commonly mediated by canonical NICD-CSL or NICD1-RBPjκ complex signaling^22^. MYC, MYB, and RARα/β/γ are well-known transcription factors (TF) that regulate *NOTCH1* transcription, as validated by chromatin immunoprecipitation (ChIP) in the GeneCards database^9,23^, but the association of this NOTCH1-related TFs with SACC lung metastasis is lacking. Conversely, *MYC* is also a common target gene of MYB and NOTCH1^24,25^. This study addresses whether ATRA directly regulates the NOTCH1 pathway and how to coordinate MYB, MYC, and NOTCH1 pathway in advanced SACC. Some findings indeed demonstrated that ATRA might induce NICD1 activation in SACC cells but has the opposite effect in glioblastoma and breast cancer cells^26,27^. Accordingly, we first propose the dual role for NICD1 in SACC, as follows: ATRA-induced upregulation of NICD1 and downregulation of MYB or MYC through RARα alters SACC cell fate differentiation and further inhibits tumor cell malignant progression. Currently, research on this topic is very limited, but this hypothesis was well-confirmed in our experimental system.

Considering the above issues, our work focused on exploring the relationships of RA signaling with *MYB* and *NOTCH1* at the single-cell level by single-cell RNA sequencing (scRNA-seq) of samples from patients without *MYB* fusions or *NOTCH1* mutations, and the results have potential therapeutic implications for SACC patients with lung metastasis.

## Materials and methods

### Ethics statement

Specimens were collected from 79 patients with SACC at Beijing Tongren Hospital; six samples from two patients were collected for single-cell sequencing, one sample was collected for ChIP-seq, ten samples were collected from five patients (matched adjacent normal tissues) for RNA-seq, and 108 samples were collected from 71 patients (37 of them matched adjacent normal tissues) for IHC. The clinicopathological features of the sampled patients are presented in Supplementary Table 1, and some information about mutations is provided in Supplementary Fig. 1. This research was approved by the Medical Ethics Committee of Beijing Tongren Hospital (Ethics approval number: TREKY2020–021) and was performed with the informed consent of the patients. Animal experiments were approved by the Animal Ethics Committee of Capital Medical University (Ethics approval number: AEEI-2020–144) and performed according to the general rules of the Capital Medical University Laboratory Animal Center.

### IHC analysis

Paraffin-embedded tissue sections (4-µm thickness) were deparaffinized in fresh xylene and subjected to antigen retrieval. Sections were then incubated overnight at 4 °C with the indicated primary antibodies. Horseradish peroxidase (HRP) activity was detected by a PV two-step IHC kit according to the manufacturer’s instructions. Rabbit or mouse monoclonal IgG was used as the negative control. Images were acquired using a Leica light microscope.

The IHC score was determined by two senior clinical pathologists. Parameter A was acquired by grading the percentage of positive cells as 1 (≤25%), 2 (26–50%), 3 (51–75%), and 4 (>75%). Parameter B represents the staining intensity, which was scored as 0 (negative, −), 1 (weak, +), 2 (intermediate, ++), or 3 (strong, +++). Then, the IHC score was calculated by multiplying A and B, and the resulting score ranged from 0 to 12^28,29^.

Details of important materials used in the study, including antibodies, drugs for in vivo and in vitro experiments, and instruments are provided in Supplementary Table 4.

### Cells

The human SACC cell lines SACC-LM (high invasiveness) and SACC-83 (poor invasiveness, RRID: CVCL_H589) were obtained from the Peking University Hospital of Stomatology. The cells were cultured in RPMI 1640 supplemented with 10% fetal bovine serum (FBS) and 1% penicillin–streptomycin solution (Invitrogen). Cell line authentication was performed by short tandem repeat (STR) PCR. All cultures were demonstrated to be negative for mycoplasma.

### Plasmid construction and transfection

Efficient and experimentally validated shRNA sequences for target genes were selected on the Sigma website (RRID: SCR_008988); these sequences are shown in Supplementary Table 5. Recombinant plasmids were constructed with T4 ligase in the pLKO.1-Puro or pCDHO-Neo-CMV-3-Flag vector and then transformed into competent *E. coli*. The pcDNA3.1-*NICD1*-3-Flag, pLKO.1-Puro, and pCDHO-neo-CMV-3-Flag vectors were gifts from Professor Zhang Yu-xiang, Capital Medical University Cancer Center.

The transformed plasmids and lentiviral plasmids were cotransfected into 293 T cells (RRID: CVCL_0063) using polyethyleneimine (PEI). The lentivirus-containing supernatant was collected and filtered after 48 hours. Subsequently, SACC-LM or SACC-83 cells were cultured with the collected supernatant and selected for one week with 2 μg/mL puromycin or 1 µg/mL neomycin to obtain stable *NOTCH1*-KD, *MYB*-KD, *NOTCH1*, and *MYB*-double-KD, or *MYB*-cDNA cells. In addition, SACC-LM cells stably expressing the luciferase gene (SACC-LM-luciferin cells) were generated. The knockdown efficiency was determined by qPCR and Western blot analyses. For transient transfections, 2–3 μg plasmid was combined with Lipofectamine 8000 for transfection into cells for 48–72 h, and transfection efficiency was confirmed by Western blotting.

### RNA-seq and ChIP-seq

We performed RNA-seq analysis of tissues and cells exposed to different treatments or conditions, including ATRA, *NOTCH1* and/or *MYB* knockdown, and *MYB* overexpression. The data are available in the Gene Expression Omnibus (GEO) database (GEO numbers: GSE216852 and GSE216913). RNA extracted from 1 × 10^7^ cells in 1 mL TRIZOL reagent was sent to Beijing Genomics Institute (BGI) for RNA-seq or ChIP-seq. Total cellular RNA was extracted using an RNA extraction kit and then reverse transcribed into cDNA using a reverse transcription kit. The qPCR primers are shown in Supplementary Table 6. The total RNA concentration, RNA integrity (RIN) value, 28 S/18 S ratio, and fragment size were measured using an Agilent 2100 Bioanalyzer. All samples that met the quality standards were used for library construction and sequencing. BGI used Illumina sequencing technology and developed the Dr.Tom system for further sequencing analysis. ChIP-seq was performed on a sample with the *MYB-NFIB* fusion according to the kit instructions. Fresh tissue samples were homogenized and crosslinked with 1% formaldehyde. Extracted DNA was fragmented by ultrasonication. The supernatant was collected and divided into three, for use as the input, IgG and MYB groups. Anti-MYB antibody (5 μg) was used for immunoprecipitation. The DNA fragments isolated from the immune complex were purified by a DNA purification kit and then analyzed by BGI. Peak values were acquired in a 2-kb region. Data processing and gene annotation were performed via the Dr.Tom system.

### Western blot analysis

Protein was extracted from cell lysates with radioimmunoprecipitation assay (RIPA) buffer. After separation by 8 or 10% sodium dodecyl sulfate-polyacrylamide gel electrophoresis (SDS–PAGE), the proteins were transferred onto polyvinylidene fluoride (PVDF) membranes. The membranes were blocked with 5% milk in TBS/Tween 20 and then incubated with primary antibodies overnight at 4 °C. Antibody information is available in Supplementary Table 4.

### Cell growth and invasion assays

For growth assays, 3000 cells/well were plated in a 96-well plate, and cell proliferation was detected at 24, 48, and 72 h with a CCK-8 assay. Cell invasion was determined using 24-well BD Corning™ cell culture inserts with 8-µm pores according to the manufacturer’s protocol. Transwell membranes coated with Matrigel® were used to evaluate the invasive ability of SACC-83-vector, SACC-83-*NOTCH1*-KD, SACC-LM-vector, and SACC-LM-*MYB*-KD cells. Cells in eight random fields of view per membrane from three chambers were counted. All experiments were repeated three times.

### Co-IP

Interactions between MYB and NICD1 or MYC were determined by Co-IP. The lysate from 1 × 10^7^ cells was precleared by 1 h incubation with 20 µL protein A/G beads. Then, after separating the protein A/G beads, the samples were incubated with specific antibodies (detailed in Supplementary Table 4) or control IgG at 4 °C overnight. After three washes with lysis buffer, the coprecipitated proteins were obtained and analyzed by Western blotting.

### Animal experiments

Male BALB/c-nude mice aged 4–6 weeks were purchased from Huafukang Biotechnology, Beijing. The lung metastasis mouse model was generated by the tail vein injection of 1 × 10^6^ SACC cells with gain or loss and analyzed (five per group) after 2 or 4 weeks, respectively. In addition, 1 × 10^5^ cells were injected intraventricularly and sampled after 4 weeks. At the endpoint of the experiment, the mice were humanely sacrificed according to laboratory rules. Whole lung tissue was resected for HE staining, and the number of metastatic lung nodules greater than 100 µm in diameter was counted. Drugs, including vehicle (10% DMSO + 30% PEG300 + 5% Tween 80 + 45% saline), 10 mg/kg DAPT, 5–10 mg/kg ATRA, or 10 mg/kg DAPT + 5 mg/kg ATRA, were administered by gavage. On days 1, 14, and 21, mice were imaged after intraperitoneal injection of 150 µg/ml d-luciferin, and the fluorescence intensity was measured with a Lumina II (PerkinElmer) imaging system.

### Single-cell sorting, t-distributed stochastic neighbor embedding (t-SNE), and cell annotation

Cells from fresh tumor tissues were isolated for the preparation of single-cell suspensions via the Chromium^TM^ Single Cell 3’ Solution technique, and then analyzed by BioMiao Biological Technology Co., Ltd. (Beijing). Raw data (150–200 Gb) were obtained from six samples. The following numbers of high-quality cells were obtained after filtering: 8124 cells in sample AP (primary paracancerous tissue); 6054 cells in sample A (primary cancer tissue); 8014 cells in sample F (matched normal lung tissue); and 7950, 13,257, and 7729 cells in samples A1, B1, and C1 (lung metastases), respectively. After log normalization and dimensionality reduction, the cells were classified into 25 clusters based on available differential gene expression data via a t-SNE algorithm in Seurat. We defined 25 cell types based on marker gene expression as suggested by BLUEPRINTENCODE, the Human Primary Cell Atlas data, and the literature (Supplementary Figs. 2, 3 and Supplementary Table 2). Additionally, a histogram was drawn to visualize the abundance of different clusters among different samples (Supplementary Fig. 2). Data has been uploaded to the public repository archival site (GEO number: GSE216852)

### Single-cell RNA velocity and pseudotime analysis

RNA velocity was performed using scVelo (0.2.4) and velocyto (0.17) in python software^30^. Pseudotime trajectory analysis was performed in Monocle 2 with expression data, barcode data, and gene ID data. After the RDS file generated from the data files was imported into R, we normalized the data, performed dimensionality reduction, and ranked the top 100 genes by *q* value. Genes at branch points were analyzed.

### Data visualization

Balloon plots, violin plots, and heatmaps analysis results were generated with the R (version 4.0.2, RRID: SCR_001905) package Monocle 2.0, while heatmaps of differential gene expression data were generated with GraphPad Prism 8 (San Diego, CA, USA, RRID: SCR_002798) or Dr.Tom (BGI). PPI analysis was performed via the STRING website (RRID: SCR_005223). The crystal structures of MYB (200-500) and MYC (100-300) were predicted with AlphaFold2. Interactive docking of protein–protein complexes was performed in the protein docking server (ZDOCK server) and visualized with PyMOL 2.5 (RRID: SCR_000305).

### Statistical analysis

GraphPad 8.0 (San Diego, CA, USA, RRID: SCR_002798) was utilized for statistical analysis. The paired two-tailed Student’s *t*-test was used for the analysis of the results from cell growth, cell invasion, colony formation, and qPCR experiments. The Mann–Whitney *U*-test was used to compare fluorescence intensity, the number of pulmonary metastatic nodules, the sequencing data of lung metastasis tissue, and the IHC results. *p* < 0.05 (*), *p* < 0.01 (**), and *p* < 0.001 (***) were considered to indicate significant differences.

## Results

### Cell composition characteristics of SACC primary tumors and lung metastases as determined by scRNA-seq

**Fig. 1:**
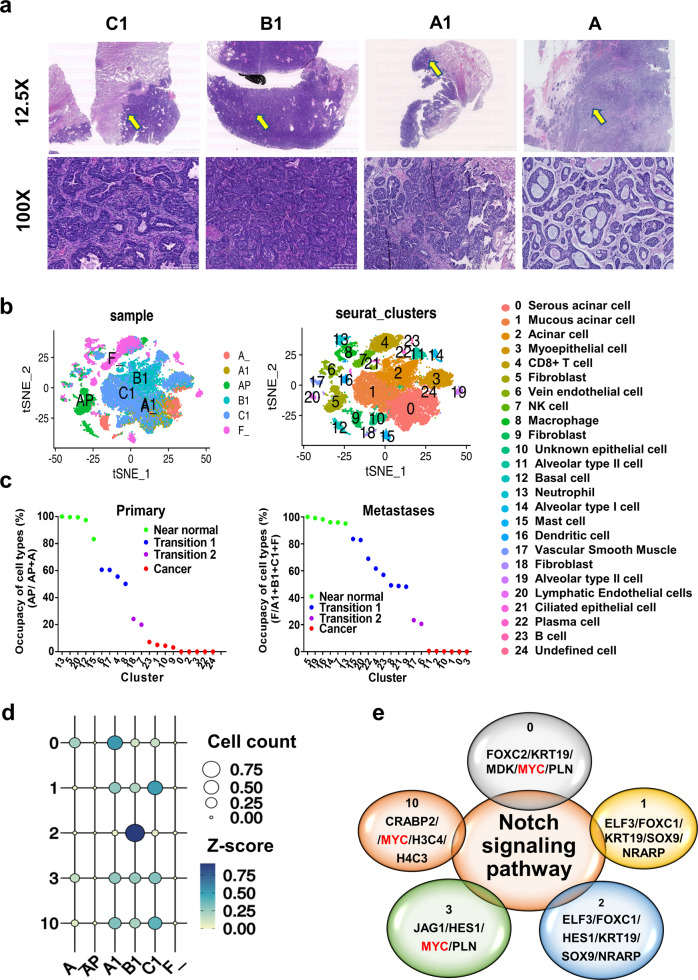
Cancer cell type definition and GO enrichment analysis based on scRNA-seq data. **a** Images of HE staining show a primary SACC lesion (A) and lung metastases in the right middle lobe (B1) and the right lower lobe (A1 and C1) at 12.5× and 100× magnification. **b** t-SNE plots of six sample types (left) and all cells (right) led to the identification of 25 cell clusters based on cellular identity, and all cell types are defined by known marker genes (see Supplementary Table 2). **c** The statistical plots show the normal tissue occupancy for each cell type (see the formula in the Results) in the primary lesion (left) and metastases (right). These data were used to divide the cells into four stages: near-normal (>80%), transition 1 (50–75%), transition 2 (15–40%), and cancer (<14%). **d** The bubble plot indicates the number of cancer-stage cells (cells in clusters 0, 1, 2, 3, and 10) in the six samples. **e** The Venn diagram shows the predominant DEGs in the cancer-stage cell clusters (clusters 0, 1, 2, 3, and 10) and the shared enriched GO term: the Notch signaling pathway.

To explore the differences in cellular composition between the microenvironments of primary tumors and lung metastases, 10× Genomics scRNA-seq was performed using primary cancerous tissues (A) and matched adjacent normal tissues (AP) from the tongue root glands and three lung metastases (A1, B1, and C1) originating from the epiglottis and matched adjacent normal lung tissues (F) (patient characteristics are presented in Supplementary Fig. 1 and Supplementary Table 1). The histopathological features were observed at 12.5× and 100× magnification (Fig. 1a). The primary SACC sample showed a predominantly tubular and solid pattern (>30%), the right lung metastasis in the middle lobe (0.4 cm, B1) exhibited a predominantly tubular pattern (tubular area ≈ 80%, solid area ≈ 20%), and the other two metastases in the lower lobe (A1, 1.3 × 1 × 0.5 cm; C1, 1.5 × 1 × 0.7 cm) showed a predominantly solid component of ~60% (A1) and 80% (C1). The A1 and C1 metastases with a predominantly solid structure should be more aggressive than the B1 metastasis and the primary tumor (A).

An estimated 51,128 single cells were captured and independently clustered into 25 distinct cell types based on their characteristic gene expression profiles (Fig. 1b). Cell types were defined in Supplementary Figs. 2, 3 and Supplementary Table 2^31,32^. In total, 17 of the 25 cell clusters were shared between the primary tumor (A) and metastases (A1, B1, and C1). However, several organ-specific cell types were observed in the lung tissues; these included basal cells (cluster 12), fibroblasts (cluster 18), undefined cells (cluster 24) in oral tissues, and alveolar type I cells (cluster 14), alveolar type II cells (cluster 11 and 19), dendritic cells (cluster 16), and ciliated epithelial cells (cluster 21). We noted differences in cell clusters between solid and tubular tumors; for example, serous acinar cells (cluster 0) were most enriched in the primary lesion (A) and metastasis A1, whereas mucous acinar cells (cluster 1) were most enriched in metastasis C1, and acinar cells (cluster 2) were most enriched in metastasis B1 (Supplementary Fig. 2a).

### The Notch signaling pathway is prevalent in the defined cancer cell clusters

Before performing an in-depth functional investigation, we first distinguished malignant and nonmalignant cells by calculating the normal tissue occupancy for each cell type as follows: primary tumor tissue frequency = each cluster ratio (AP)/sum of the cluster ratios (A + AP) *100%; metastasis tissue frequency = each cluster ratio (F)/sum of the cluster ratios (A1 + B1 + C1 + F) *100%. A frequency of 100 or 0% means that these cell clusters are found exclusively in normal tissue or cancerous tissues, respectively. Based on the frequency value, we classified the 25 cell clusters as belonging to four main stages: (1) >80%, near-normal cell stage (clusters 13, 5, 20, 12, and 15 in primary tumors and clusters 5, 19, 16, 14, 7, and 13 in metastases); (2) 50 to 75%, cancer transition 1 stage (clusters 6, 17, 4, and 8 in primary tumors and clusters 15, 20, 22, 4, 23, 8, 21, and 9 in metastases); (3) 15 to 40%, cancer transition 2 stage (cluster 18 and 7 in primary tumors and clusters 17 and 6 in metastases); and (4) <14%, cancer stage (clusters 23, 1, 10, 9, 0, 2, 3, 22, and 24 in primary tumors and clusters 11, 2, 10, 1, 0, and 3 in metastases) (Fig. 1c). The frequency value at stage 1 or 4 should be better in distinguishing the malignant and nonmalignant cells than that at other stages. Notably, serous acinar cells (cluster 0), mucous acinar cells (cluster 1), acinar cells (clusters 2), myoepithelial cells (cluster 3), and unknown epithelial cells (cluster 10), all of which are suggested in the literature to be the primary constituents of cancer tissues^33,34^, were found in both metastatic and primary cancer samples (Fig. 1d). We therefore further investigated the functional characteristics of these cell clusters.

Gene Ontology (GO) functional analysis was performed, and examination of the intersecting biological process (BP) terms enriched in all five cancer cell clusters identified the Notch signaling pathway; the core genes of this pathway, including *MYC*, are highlighted in red (Fig. 1e). GO enrichment analysis showed that the genes enriched in cluster 10, which had the lowest cell count of the five cancer cell clusters, were related mainly to the cell cycle and mitosis, implying that cluster 10 consists of cells with high proliferative activity (Supplementary Fig. 4). These cells may be metastatic stem cells, which promote metastasis when the proliferation rate is high, as hypothesized in the literature^35^.

### Signature genes in the putative metastasis-associated clusters and cancer stem cells are linked to *NOTCH1*

**Fig. 2:**
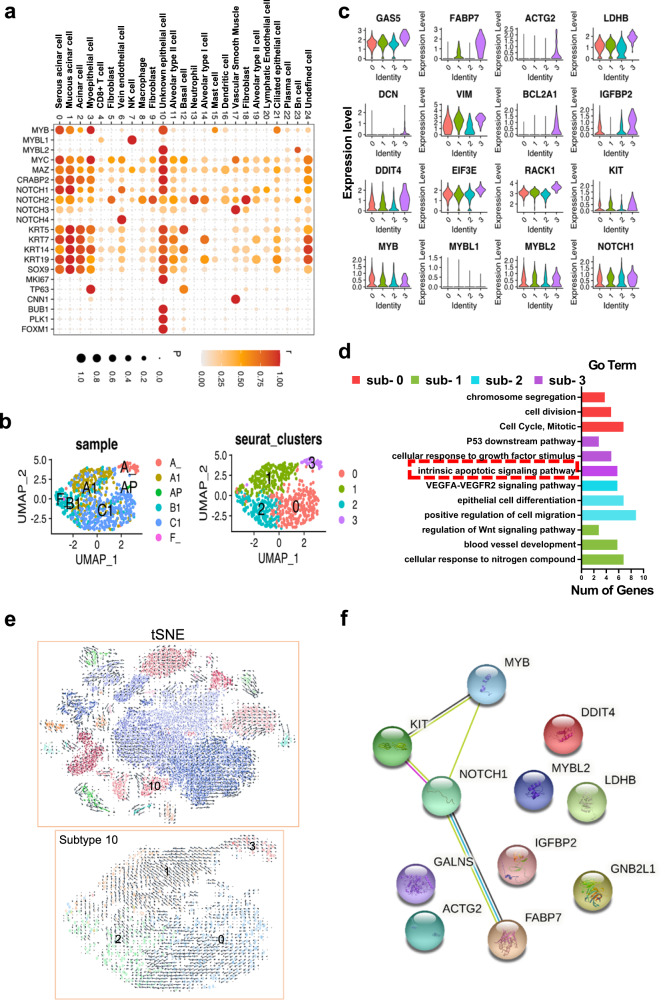
Characterizing metastasis-associated clusters or cancer stem cells. **a** The dot plot shows marker genes likely related to SACC stemness and metastasis, as reported in the literature, that were enriched predominantly in cluster 10. **b** The t-SNE visualization of novel subtypes in cluster 10 shows the distribution in samples (left) and four novel subsets (right). **c** Violin plots of signature genes, including the top 12 marker genes, as well as *MYB, MYBL1, MYBL2*, and *NOTCH1* in all the subtypes from cluster 10. **d** GO analysis of all subtypes; subtype 3, which is enriched in genes related to the antiapoptotic pathway, is highlighted. **e** RNA velocity analysis of all cells and all subtypes by Velocyto soft. The arrow in the plot indicates the direction of differentiation, and the length of the arrow indicates the speed of differentiation (a longer arrow indicates a faster speed). **f** PPI analysis showing the known interactions, *MYB/MYBL2*, *NOTCH1*, and the top eight marker genes.

We wondered whether cluster 10 contains cancer stem cells closely associated with the lung metastasis of SACC. First, we investigated the expression of selected marker genes reportedly related to SACC stemness and metastasis in all 25 cell clusters^31^. The resulting dot plot confirmed the enrichment of some unique cell proliferation-related genes, such as *MKI67, MYBL2, BUB1, PLK1*, and *FOXM1*, in cluster 10 (Fig. 2a and Supplementary Fig. 3b). Second, we divided cluster 10 into 4 subtypes to further characterize the cell subtypes via Seurat clustering. Unlike the other three subtypes, subtype 3 was mainly distributed in the primary lesion (A) (Fig. 2b) and exhibited higher expression of *KIT*, fatty acid-binding protein 7 (*FABP7*)*, LDHB, BCL2A1*, *RPS3*, *IGFBP2*, etc. but not of *MYB, NOTCH1*, or *MYBL2* (Fig. 2c and Supplementary Fig. 5a). Moreover, in contrast to the biological function terms enriched in the other three subtypes, the terms enriched by the differentially expressed genes (DEGs) in subtype 3 were mainly related to apoptosis resistance-related pathways (Fig. 2d). Based on data from the literature, the cells in subtype 3 were presumed to be “seed” cancer cells associated with metastasis, i.e., cancer stem cells^36^.

To track the direction of stem cell differentiation, we performed RNA velocity and pseudotime analysis of the four subtypes. RNA velocity analysis indicated that cluster 10 and subtype 3 had a tendency to differentiate into the other cells (Fig. 2e). The trajectory visualized with Monocle 2 clearly showed that the roots and branches of the primary tumor samples and subtype 3 had similar shapes (Supplementary Fig. 5b, highlighted in red boxes), confirming that subtype 3 originated from the primary tumor. Subsequently, heatmaps were generated to compare DEGs among the four subtypes (Supplementary Fig. 6a), which revealed that proliferation-related genes such as *RP53* and *RPL29* were mainly enriched in subtype 3, implicating these gene products in the promotion of metastasis. Some unique cell proliferation-related genes and the top 20 signature genes for subtype 3 are shown in Supplementary Fig. 6b; these genes were predicted to directly interact with NOTCH1 or MYB in the protein–protein interaction (PPI) analysis (Fig. 2f and Supplementary Fig. 6c). Notably, *MYB, KIT*, and *FABP7* (the top-ranked signature genes) directly interact with *NOTCH1*, and these interactions have been reported to be involved in regulating cancer stem cell differentiation^37–39^. The other signature genes did not show reciprocal associations with each other (Fig. 2f).

### Both MYB and NOTCH1 contribute to the lung metastasis of SACC

**Fig. 3:**
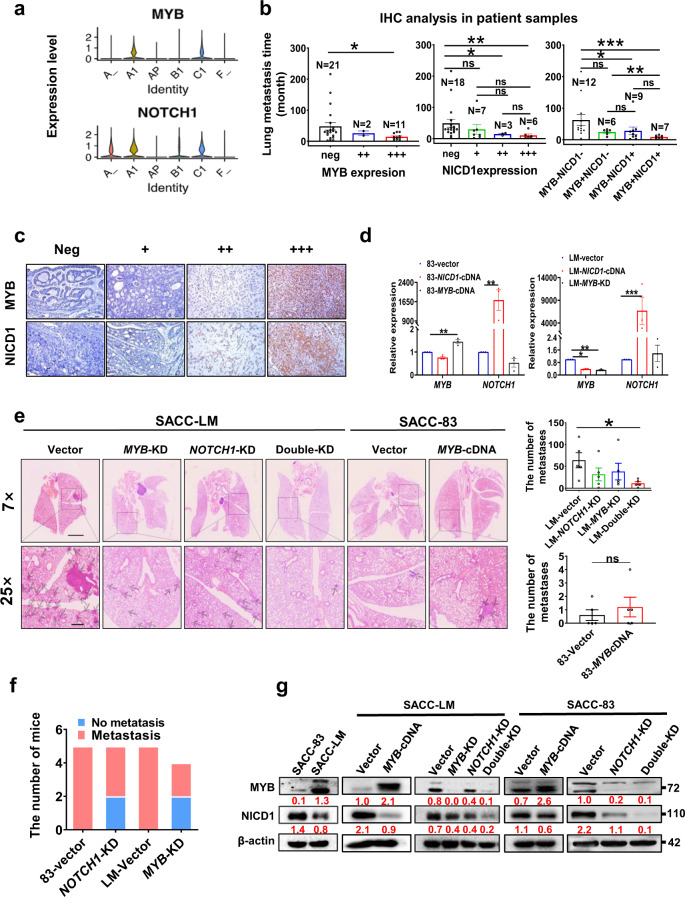
Abnormal expression of both NOTCH1 and MYB cooperatively promotes the lung metastasis of SACC. **a** Violin plot depicting the expression levels of *MYB* and *NOTCH1* in six samples. **b** Statistical analysis of the association between the time to lung metastasis in 34 patients and MYB or/and NICD1 protein levels based on IHC staining. **c** Representative IHC images of MYB and NOTCH1 expression in lung metastatic lesions from patients (*n* = 34). **d** qPCR analysis of the efficiency of *NOTCH1* or *MYB* overexpression or *MYB* knockdown in two SACC cell lines. **e** Representative HE images of lung tissues from mice with metastases at 2 or 4 weeks after tail vein injection with different cell lines (7× and 25× magnification). The statistical analysis of the number of metastases in lung tissues is shown on the right. **f** Statistical analysis and stacked bar chart of the number of mice with lung metastases at 4 weeks after the left ventricular injection of SACC cells (5 per group). **g** Western blot validation of MYB and NICD1 expression in SACC-83-vector, SACC-LM-vector, SACC-LM-*MYB*-cDNA, SACC-LM-*NOTCH1*-KD, SACC-LM-*MYB*-KD, SACC-LM-double-KD, SACC-83-vector, SACC-83-*MYB*-cDNA, SACC-83-*NOTCH1*-KD, and SACC-83-double-KD cells. β-Actin served as a loading control (*n* = 3). The *p* values in (**b**) and (**e**) were calculated using the two-tailed Mann–Whitney *U-*test, and those in (**c**) were calculated using the paired two-tailed Student’s *t*-test. Data were presented as the mean ± SEM. *p* < 0.05 (*), *p* < 0.01 (**), *p* < 0.001 (***), and *p* > 0.05 (n.s.).

Next, we validated the roles of MYB and NOTCH1 in promoting the lung metastasis of SACC. The scRNA-seq data indicated that *MYB* expression was elevated in metastatic SACC lesions compared to primary SACC lesions; however, *NOTCH1* expression was elevated in both metastatic and primary lesions (Fig. 3a). IHC analysis of 34 SACC cases (Supplementary Table 1) revealed that MYB-positive (+++) and NICD1-positive (++ and +++) cases had a significantly shorter time to lung metastasis than the corresponding negative cases. Moreover, seven cases with positive coexpression of MYB (+) and NICD1 (+) had shorter times to lung metastasis than those negative for MYB and NICD1 or positive for a single marker (Fig. 3b, c). The IHC score is defined in the Materials and Methods.

For cell models, the highly metastatic SACC-LM cell line was utilized; this cell line originated from a lung metastasis of the poorly metastatic SACC-83 cell line in nude mice. Western blot analysis demonstrated higher levels of MYB and lower levels of NOTCH1 in SACC-LM cells than in SACC-83 cells (Fig. 3g), and RNA-seq validated that neither cell line has an *MYB-NFIB* fusion or a *NOTCH1* mutation (Supplementary Fig. 7). Therefore, we successfully constructed different stable overexpression (cDNA) or knockdown (KD) cell models [SACC-83-*MYB*-cDNA, SACC-83-*NOTCH1*-KD, SACC-83-*MYB-NOTCH1*-double-KD (SACC-83-double-KD), SACC-LM-*MYB*-KD, SACC-LM-*NOTCH1*-KD, SACC-LM-*MYB*-cDNA, and SACC-LM-*MYB-NOTCH1*-double-KD (SACC-LM-double-KD)] and validated these models by quantitative real-time PCR (qPCR) or Western blot analysis (Fig. 3d, g). In line with the results of previous studies, the knockdown of *NOTCH1* or *MYB* significantly reduced cell growth, colony formation, and cell invasion^40,41^ (Supplementary Fig. 8a).

In the in vivo experiment, 1 × 10^6^ SACC-LM-vector, SACC-LM-KD, SACC-83-vector, and SACC-83-*MYB*-cDNA cells were injected into BALB/c-nude mice via the tail vein; after 2 (SACC-LM lines) or 4 (SACC-83 lines) weeks, whole lung tissue was resected and examined by hematoxylin–eosin (HE) staining. Lung tissues from the SACC-LM-double-KD group had significantly fewer and smaller metastatic foci than those in the SACC-LM-vector, SACC-LM-*MYB*-KD, or SACC-LM-*NOTCH1*-KD group (Fig. 3e). The SACC-83-*MYB*-cDNA group showed an increasing trend in lung metastatic foci or tumor size compared to the SACC-83-vector group (Fig. 3e). NOTCH1 expression at both the RNA level and protein level was higher in the SACC-83 cell line than in the SACC-LM cell line, so we hypothesized that it might be an early driver gene in the SACC-83 cell line. In contrast, MYB expression was higher in the SACC-LM cell line than in the SACC-83 cell line, and therefore it was hypothesized to be an early driver gene in the SACC-LM cell line. Additionally, due to the low metastasis rate of SACC-83 cells, we injected mice with 1 × 10^5^ vector or KD cells via the left ventricle to mimic blood metastasis and found a significant decrease in lung metastases in the SACC-83-*NOTCH1*-KD and SACC-LM-*MYB*-KD groups (Fig. 3f and Supplementary Fig. 8b, c). These data support the hypothesis that the abnormal expression of both NOTCH1 and MYB plays an early role in driving the lung metastasis of SACC.

### Activation of the ‘MYC_TARGETS_V2” gene set is associated with the lung metastasis of SACC

To investigate metastasis-related Kyoto Encyclopedia of Genes and Genomes (KEGG) pathways, we first identified the overlapping KEGG pathways for the subtype 3, SACC-LM cell, and SACC-83 cell groups based on RNA-seq data. The “MYC_TARGETS_V2” gene set was present in both the subtype 3 and SACC-LM cell groups, while the “MYC_TARGETS_V1” gene set was present exclusively in the SACC-83 cell group. Unexpectedly, the “MYC_TARGETS_V2” gene set was also enriched in our MYB-ChIP-seq data from a sample harboring the *MYB-NFIB* fusion, and we identified 150 target genes of MYB (Supplementary Table 3). These findings suggest that the MYB transcription factor ultimately promotes the expression of the “MYC_TARGETS_V2” hallmark gene set.

**Fig. 4:**
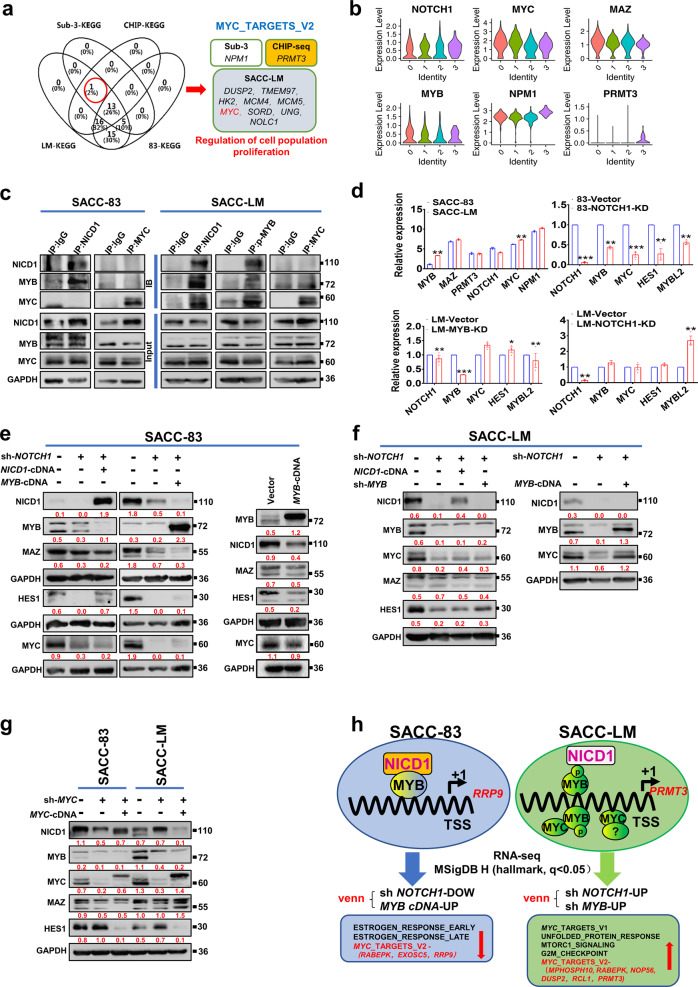
The NICD1–MYB complex targets the “MYC_TARGETS_V2” gene set to initiate the lung metastasis of SACC. **a** Venn diagram showing the number of overlapping genes identified by GSEA (*q* < 0.05) in SACC-LM cells, SACC-83 cells, subtype 3, and ChIP-seq data. Marker genes are listed on the right. **b** Violin plots of the expression of signature genes in the “MYC_TARGETS_V2” gene set for the four subtypes of cluster 10. **c** Endogenous Co-IP analysis of NICD1, MYB, and MYC in SACC-83 and SACC-LM cells. **d** RNA-seq data showing the expression levels of the screened “MYC_TARGETS_V2” gene set in SACC-LM cells compared to SACC-83 cells (left, upper). qPCR analysis of genes related to Notch signaling after knockdown of *NOTCH1* or *MYB* in SACC cells. *p* values were calculated using the paired two-tailed Student’s *t*-test. Data were presented as the mean ± SEM. *p* < 0.05 (*), *p* < 0.01 (**), *p* < 0.001 (***). **e**, **f** Western blot analysis of the change in NOTCH1-related proteins in SACC-83-*MYB*-cDNA cells or after rescuing NICD1 or MYB expression in SACC-83-*NOTCH1*-KD or SACC-LM-*NOTCH1*-KD cells. **g** Western blot analysis of the indicated proteins upon knockdown or rescue of MYC expression in SACC-83 or SACC-LM cells. **h** Schematic diagram visualizing two different mechanisms by which NICD1 recruits MYB to target the “MYC_TARGETS_V2” gene set in poorly metastatic SACC-83 cells and recruits the pMYB–MYC, pMYB–MYC or unknown MYC complex to target the “MYC_TARGETS_V2” gene set in highly metastatic SACC-LM cells.

Notably, the specific signature genes in the “MYC_TARGETS_V2” gene set that were enriched in each sample differed. For example, *DUSP2, TMEM97, HK2, MCM4, MCM5, MYC, SORD, UNG*, and *NOLC1* were enriched mainly in SACC-LM cells, *NPM1* was enriched mainly in single-cell samples without *MYB* fusion, and *PRMT3* was enriched mainly in CHIP-seq samples with *MYB* fusion. However, these genes share a common KEGG pathway related to the regulation of cell proliferation (Fig. 4a). Consistent with these results, the signature genes *NPM1* and *PRMT3* were also enriched in subtype 3 (Fig. 4b). RNA-seq and qPCR confirmed that the higher levels of *MYB, MAZ, MYC*, and *NPM1* were higher in SACC-LM cells than in SACC-83 cells (Fig. 4d). Here, we selected oncogenic *MYC* as a representative of the “MYC_TARGETS_V2” gene set to investigate pathways and interactions through in vitro experiments.

### NICD1 recruitment of MYB to activate MYC may be an important mechanism that promotes the lung metastasis of SACC cells

Western blot analyses (Fig. 3g) showed that *NOTCH1* knockdown induced the downregulation of MYB in both SACC-LM and SACC-83 cells. MYB expression in SACC-83-double-KD cells was similar to that in SACC-83-*NOTCH1*-KD cells; conversely, *MYB* knockdown did not significantly downregulate NICD1 in SACC-LM cells in three replicate tests. These data suggest that MYB is a downstream effector of NOTCH1 in SACC progression.

NOTCH1, which directly targets MYC, is considered a common noncanonical Notch signal mediator and is closely associated with lymphoma tumorigenesis and SACC progression^18,42^. Therefore, we hypothesized that NICD1 recruits MYB to enhance MYC expression, thereby promoting metastasis. The endogenous Co-IP assay results showed the presence of the NICD1–MYB complex in poorly metastatic cells (SACC-83 cells); however, a NICD1–phosphorylated MYB–MYC complex was detected in highly metastatic cells (SACC-LM cells) (Fig. 4c). We found that NICD1 and MYB exhibited a mutually exclusive exogenous gene expression pattern, which limited further validation via exogenous IP assays (Fig. 4e, f). The mechanism underlying this mutual exclusivity is not clear. However, AlphaFold2 and protein docking (ZDOCK) analysis helped validate the presence of specific binding sites between NICD1 and MYB and between MYB and MYC within the transcriptional activation domain of MYB (Supplementary Fig. 9).

Next, we investigated this pathway via Western blot analysis. Knockdown of *NOTCH1* (sh-*NOTCH1*) in SACC-83 and SACC-LM cells was shown to downregulate MYC, as well as MYB, MAZ, and HES1. MAZ was reported to act as an active transcription factor of MYB^43^. Paradoxically, after supplementation with *NICD1*-cDNA, HES1 protein expression was rescued in SACC-83 cells but not in SACC-LM cells (Fig. 4e, f). However, after the introduction of *MYB-*cDNA, MYC protein expression was rescued in SACC-LM cells (Fig. 4f); MYB overexpression caused the downregulation of NICD1, MAZ, HES1, and MYC in SACC-83 cells (Fig. 4e). In contrast, *MYC* knockdown in both SACC-83 and SACC-LM cells decreased the levels of MYB but not those of MAZ, NICD1, or HES1, and these effects could not be rescued by *MYC*-cDNA (Fig. 4g). RNA-seq analysis indicated that the changes in protein and RNA levels were consistent in SACC-83 cells but not in SACC-LM cells (Fig. 4d). Genes downregulated by *NOTCH1-*KD and upregulated by *MYB-*cDNA in SACC-83 cells, and specifically certain signature genes (*RABEPK, EXOSC5*, and *RRP9*), were coenriched in the “MYC_TARGETS_V2” gene set, suggesting that the “MYC_TARGETS_V2” gene set is controlled by both NOTCH1 and MYB in SACC-83 cells. Surprisingly, genes upregulated by sh-*NOTCH1*, sh-*MYB*, or even the double-KD in SACC-LM cells, including *RCL1, PRMT3, MPHOSPH10, RABEPK, NOP56*, and *DUSP2*, were also detected within the “MYC_TARGETS_V2” gene set (Fig. 4h). These results indicate that “MYC_TARGETS_V2” is an alternative pathway that is used upon loss of the NOTCH1–MYB pathway in SACC-LM cells.

### Abnormal NOTCH1 expression activates RA signaling to downregulate MYB and MYC

**Fig. 5:**
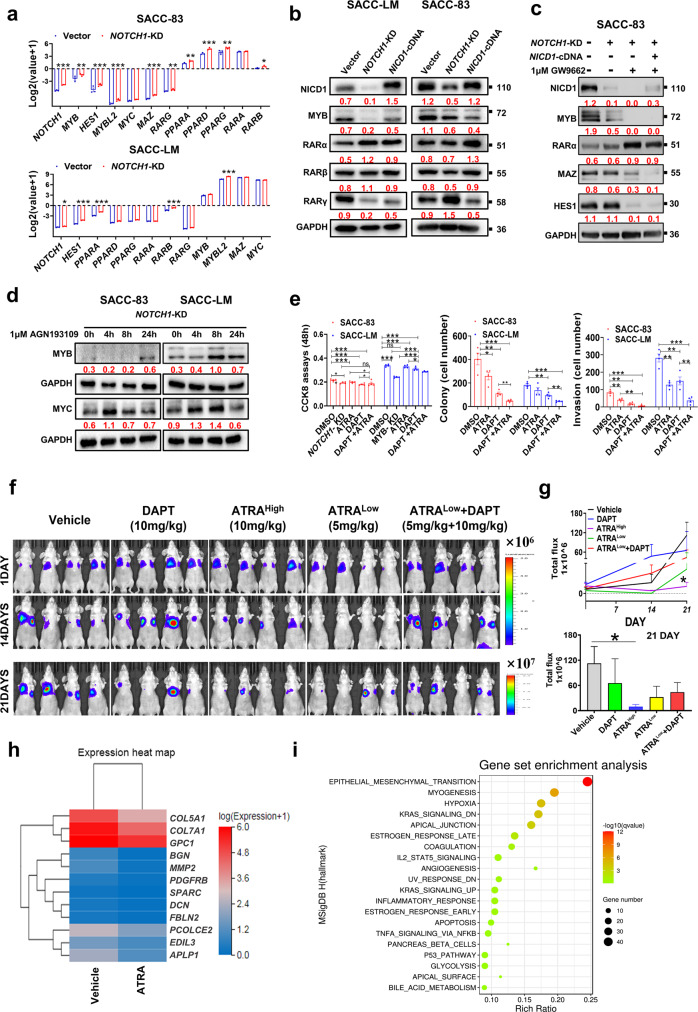
*NOTCH1* knockdown triggers RA signaling to downregulate MYB, which inhibits the lung metastasis of SACC. **a** Screening of RNA-seq data (three replicates per group) for genes that are up- and down-regulated upon *NOTCH1* knockdown in SACC-83 and SACC-LM cells. Data analysis was performed via the Dr.Tom system (BGI). **b** Western blot validation of the changes in levels of MYB and RARs upon knockdown and rescue of *NOTCH1* in SACC-83 cells and SACC-LM cells. **c** Western blot analysis of the changes in levels of targeted proteins in SACC-83 cells after treatment with 1 μM GW9662 (PPARγ inhibitor). **d** After *NOTCH1* knockdown in two SACC cell lines and treatment with 1 μM AGN193109 (RAR antagonist), Western blot analysis was performed to evaluate the time-dependent changes in MYB and MYC expression. **e** Statistical analysis of cell proliferation, colony formation, and invasion assay results after treatment with ATRA (1 μM), DAPT (20 μM), or ATRA and DAPT or after *NOTCH1* or *MYB* knockdown. Each experiment was repeated four times. **f** Luciferase imaging of lung metastatic lesions in nude mice after the intravenous injection of SACC-LM-luciferin cells and treatment with ATRA (5 mg/kg or 10 mg/kg), DAPT (10 mg/kg), or both (5 mg/kg ATRA; 10 mg/kg DAPT) (*n* = 4/group). **g** Statistical analysis of fluorescence intensity values of lung metastatic lesions for each group on days 1, 14, and 21, with a focus on day 21. **h** DEGs (FC >2 and *p* < 0.05) are shown in the heatmap, and the GSEA results are shown (**i**) in a dot plot (*q* < 0.05). The *p* values were calculated using the paired two-tailed Student’s *t*-test (**e**) and the two-tailed Mann–Whitney *U*-test (**g**). Data were presented as the mean ± SEM. *p* < 0.05 (*), *p* < 0.01 (**), and *p* < 0.001 (***).

The failure of *NICD1-*cDNA to rescue MYB expression indicated that MYB is an indirect downstream target of NOTCH1. The RNA-seq results indicated that *NOTCH1* knockdown in SACC-83 cells downregulated the transcription factors *MYB, HES1, MYBL2, MYC*, and *MAZ* and, conversely, upregulated *PPAR*A, *PPARD, PPARG, RARA*, and *RARB*. Interestingly, *PPAR*A and *RARB* were downregulated in SACC-LM-*NOTCH1-*KD cells (Fig. 5a). The increased retinoic acid receptor (RAR) γ levels in SACC-LM-*NOTCH1*-KD cells and decreased RARγ levels in SACC-83-*NOTCH1*-KD cells were validated by Western blot analysis. Interestingly, upon overexpression of NICD1, RARγ levels were rescued in the two cell lines (Fig. 5b). These data suggest that NOTCH1 can inversely regulate RARγ expression at the protein level in both cell lines.

To investigate why MYB was downregulated, SACC-83-*NOTCH1*-KD cells were treated with 1 μM GW9662 (PPARγ inhibitor), which failed to rescue MYB, MAZ, or HES1 expression; however, treatment with 1 μM AGN193109 (RAR inhibitor) significantly rescued the expression of MYB and MYC (Fig. 5c, d) in a time-dependent manner. These findings suggest that both knockdown and rescue of NOTCH1 can induce the downregulation of MYB and MYC through the RA pathway.

ATRA, a strong inhibitor of MYB transcription, has been used for the treatment of SACC^14,15^. We hypothesized that the combination of ATRA with a NOTCH1 inhibitor would enhance the inhibition of lung metastasis. In vitro experiments showed that the combination of ATRA (1 μM) and DAPT (γ-secretase and NOTCH1 inhibitor) was superior to either ATRA (1 μM) or DAPT (20 μM) alone; the combination significantly suppressed cell proliferation (48 h), invasion (24 h), and colony formation and growth (7 days) compared to that with other treatments (Fig. 5e). Compared with DAPT alone or DAPT in combination with ATRA, a low dose of ATRA (5 mg/kg) significantly inhibited in vivo lung metastasis (Fig. 5f, g) after intragastric administration for 21 days beginning the day after the intravenous injection of SACC-LM-luciferin cells, which were used for imaging of metastatic lesions.

Downregulated genes were then identified in ATRA-treated cells compared to vehicle (control)-treated cells by RNA-seq (Fig. 5h, log2FC > 1.5, *p* < 0.05). Gene set enrichment analysis (GSEA) revealed that untreated SACC-LM-double-KD cells and ATRA-treated SACC-LM-vehicle cells were coenriched in genes mainly in the “EPITHELIAL_MESENCHYMAL_TRANSITION”, “KRAS_SIGNALING_DN”, “HYPOXIA”, “MYOGENESIS”, “ESTROGEN_RESPONSE_LATE”, and “APICAL_JUNCTION” gene sets (Fig. 5i). Obviously, double-KD of *MYB* and *NOTCH1* or treatment with ATRA reduced lung metastasis through epithelial–mesenchymal transition (EMT), which affects cell differentiation.

### The RA pathway provides surveillance for erroneous cell differentiation through a mechanism involving NOTCH1 and MYB

**Fig. 6:**
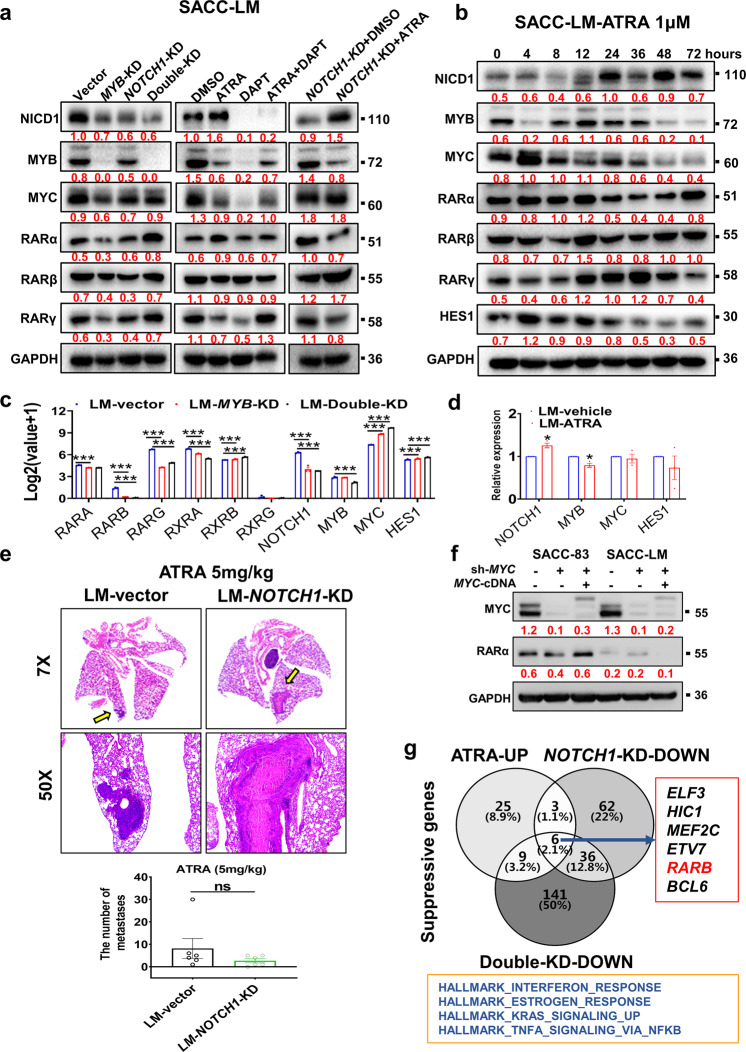
ATRA suppresses the lung metastasis of SACC in a mouse model, possibly by activating the tumor-suppressive role of NOTCH1. a Western blot analysis of RAR and MYC levels in SACC-LM cells upon knockdown of *MYB* and *NOTCH1* alone or in combination or upon treatment with ATRA (1 µM), DAPT (20 µM), or both for 48 h. The grayscale values were calculated with ImageJ 4.0. **b** Western blot analysis of the time-dependent changes in RARs and HES1 in SACC-LM cells treated with ATRA (1 µM). **c** RNA-seq data (triplicate samples for each group) showing the changes in *RAR* and *MYC* mRNA levels after treatment or knockdown. Data analysis was performed via the Dr.Tom system (BGI). **d** qPCR validation of *NOTCH1, MYB, MYC*, and *HES1* mRNA levels after ATRA treatment compared to control. **e** Representative HE images showing the lung metastases in the SACC-LM-*NOTCH1*-KD and SACC-LM-vector groups treated with ATRA (5 mg/kg) for 21 days (*n* = 6/group). The number of metastatic pulmonary nodules larger than 100 µm in diameter was calculated. **f** Western blot analysis of RARα expression upon *MYC* knockdown or rescue. **g** The Venn diagram shows the intersection of DEGs from ATRA treatment, *NOTCH1*-KD, and double-KD in SACC-LM cells to screen for NOTCH1-related genes that suppress metastasis. The grayscale values of the Western blot were calculated by ImageJ 4.0. The Western blot and qPCR experiments were repeated three times. GO enrichment analysis was performed in the Dr.com system (BGI). The *p* values were calculated using the paired two-tailed Student’s *t*-test (**d**) and the two-tailed Mann–Whitney *U*-test (**e**). Data were presented as the mean ± SEM. *p* < 0.05 (*), *p* < 0.01 (**), *p* < 0.001 (***)*, p* > 0.05 (n.s.).

We next determined how NOTCH1 and MYB activate RARs by performing Western blot and RNA-seq. In SACC-LM cells, double-KD of *MYB* and *NOTCH1* or treatment with ATRA potently upregulated RARα at the protein level but not the RNA level (Fig. 6a, c). DAPT alone (20 μM) or in combination with ATRA (1 μM) did not increase RAR levels but did markedly decrease the expression of NICD1, MYB, and MYC (Fig. 6a). Surprisingly, in SACC-LM-*NOTCH1-*KD cells, ATRA strongly increased NICD1 and RARβ levels and decreased MYB levels (Fig. 6a). Furthermore, treatment with 1 μM ATRA increased NICD1 and RARγ expression and decreased MYC, MYB, and HES1 expression in a time-dependent manner (Fig. 6b), consistent with the qPCR results (Fig. 6c, d). These data indicate that MYB or NOTCH1 may be a direct downstream target of ATRA. After 21 days of ATRA treatment (5 mg/kg), there were larger metastatic nodules in the SACC-LM-*NOTCH1-*KD group than in the vector group, but the difference in the number of metastatic lesions was not statistically significant (Fig. 6e, *p* = 0.25). Clearly, NOTCH1 is expected to have dual roles as both a tumor suppressor and an oncogene. However, RA switches the effect of the Notch1 pathway to tumor suppression.

In addition, knockdown and rescue of *MYC* in SACC-83 and SACC-LM cells did not markedly change RARα levels, verifying that MYC does not regulate RA signaling via feedback (Fig. 6f). Further experiments focused on the tumor-suppressing Notch1 pathway at the transcriptional level. Venn diagram analysis of RNA-seq data revealed six transcription factors with tumor suppressor roles; these transcription factors were affected in SACC-LM cells upon *NOTCH1* knockdown, double-KD or ATRA treatment and included *ELF3, HIC1, MEF2C, ETV7, RARB*, and *BCL6*. All the upregulated genes shared among ATRA-treated SACC-LM cells, SACC-LM-double-KD cells, and SACC-LM-*NOTCH1*-KD cells were significantly coenriched in the “INTERFERON_GAMMA_RESPONSE”, “ESTROGEN_RESPONSE_EARLY”, “INTERFERON_ALPHA_RESPONSE”, “KRAS_SIGNALING_UP”, and “TNFA_SIGNALING_VIA_NFKB” gene sets (*q* < 0.05, Fig. 6g).

### The development of SACC lung metastases may be associated with the inactivation of the RA pathway

**Fig. 7:**
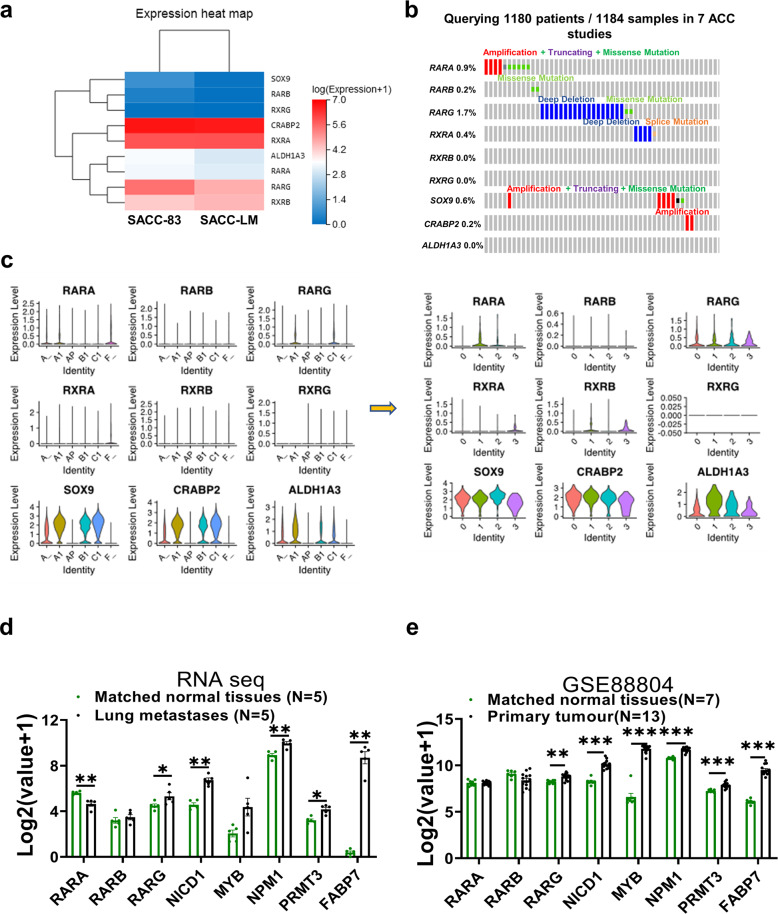
Lung metastasis in patients with SACC might partly be caused by insufficient RA signaling. **a** Heatmap of the expression of genes related to RA signaling in SACC-83 cells and SACC-LM cells, as determined by RNA-seq. **b** Mutation analysis of RA signaling molecules from the cBioPortal database in 1184 samples from 1180 patients based on seven ACC studies. **c** Violin plot of scRNA-seq analysis of the expression levels of RA signaling molecules from six tissues of two patients and four subtypes of cluster 10. **d**, **e** Statistical analysis of the mRNA levels of *MYB, NICD1, FABP7, NPM1, PRMT3*, *and RARs* in five lung metastasis samples or 13 primary tumor samples (GSE 88804) versus matched normal tissue samples. The *p* values were calculated using the two-tailed Mann–Whitney *U*-test. Data were presented as the mean ± SEM. *p* < 0.05 (*), *p* < 0.01 (**), and *p* < 0.001 (***).

**Fig. 8:**
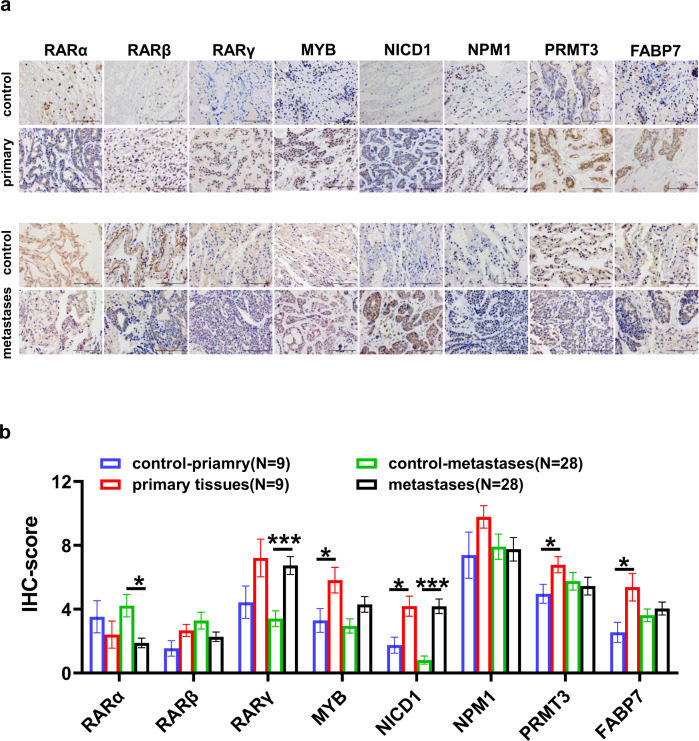
RARα shows significant downregulation in ACC metastatic lung tissues. **a** Representative images of MYB, NICD1, FABP7, NPM1, PRMT3, and RARs IHC in 28 ACC lung metastasis samples or nine primary tumor samples versus matched normal tissue samples and statistical analysis (**b**). The *p* values were calculated using the two-tailed Mann–Whitney *U*-test. Data were presented as the mean ± SEM. *p* < 0.05 (*), *p* < 0.01 (**), and *p* < 0.001 (***).

Accordingly, we wondered whether inactivation of the RA pathway occurs in patients with SACC lung metastasis. RNA-seq data for SACC-LM and SACC-83 cells indicated that the mRNA levels of RA pathway-related signaling molecules, such as *RARA, RARB, RARG, SOX9*, and *ALDH1A3*, were lower in SACC-LM cells than in SACC-83 cells (Fig. 7a). Moreover, we investigated the mutations in RARs and transporters in 1184 ACC samples and found that *RARG* (which encodes an important receptor induced by ATRA) had a deep deletion rate of 1.7% (Fig. 7b). Further single-cell analysis revealed that the expression of an ATRA-induced rate-limiting enzyme (*ALDH1A3*) and its essential transporter protein (*CRABP2*) was significantly increased in cancer stem cell clusters and cancer tissues, while the remaining RARs (those other than *RARG*) were weakly expressed (Fig. 7c). *RARA* expression was significantly downregulated in lung metastatic tissues (Fig. 7d), but not in the primary tissues (Fig. 7e, data from GSE: 88804). Subsequently, we performed IHC to examine the expression of RARs in 37 patients with SACC and detected the significant upregulation of MYB, NICD1, and RARγ in primary, and upregulation of NICD1, and RARγ, in lung metastases compared to paracancerous normal tissues. Consistently, RARα is downregulated in lung metastases (Fig. 8a, b; calculation of the IHC score is described in the Materials and Methods).

## Discussion

We identified that the Notch signaling pathway was utilized by all cancer cell clusters at the single-cell level (Fig. 1), supporting the long-held view that Notch signaling is important for SACC cell survival. Generally, Notch signals can activate differentiation programs via the canonical Notch pathways, which involve NOTCH receptors (NOTCH1–4) and ligands (Dll, Jagged1 and 2, DLK, etc.), nuclear effectors (RBPjκ, CBF-1, MAML), and canonical target bHLH gene families (*HES*, *ESR*, and *HEY*)^44–47^. By contrast, MYB, a hallmark of undifferentiated stem cells, functions by inhibiting differentiation programs and promoting stem cell proliferation^25,48^. Accordingly, NOTCH1 and MYB have opposing effects on differentiation in SACC. This knowledge could explain why MYB and NOTCH1 exhibit a mutually exclusive exogenous expression pattern in vitro (Fig. 3d, g), which limited our exogenous IP experiments. Additionally, some evidence from our study indicates that NICD1 tends to be downregulated upon MYB overexpression at both the transcript level (Fig. 3d) and protein level (Fig. 3g); the *NOTCH1* region was not identified in our MYB-ChIP-seq data (Supplementary Table 3). These observations do not support the hypothesis that MYB activates NOTCH1 transcription in our study.

Importantly, our experiments found that although NOTCH1 and MYB exhibit a mutually exclusive expression pattern when those factors are overexpressed, MYB establishes the link between NICD1 and the target MYC; this finding has not been previously reported in the literature. Through GSEA, we found that the lung metastasis of SACC is closely associated with the “MYC_TARGETS_V2” gene set, which was enriched mainly in cancer stem cell clusters and metastatic cell lines (Fig. 4a). The “MYC_TARGETS_V2” gene set is a subgroup of the genes regulated by the transcription factor MYC. “HALLMARK_MYC_TARGETS_V1” and “HALLMARK_MYC_TARGETS_V2” were identified by gene set variation analysis (GSVA)^49^. Amy Schulze et al. reported that the MYC target score was related to breast cancer aggressiveness and metastasis^50^.

Subsequently, we found that MYB activated the “MYC_TARGETS_V2” gene set through the NICD–MYB or NICD–pMYB–MYC complex (Fig. 4c). AlphaFold2 crystal structure predictions support a physical NICD1–MYB interaction (Supplementary Fig. 9). Renata Ferrarotto et al. reported that NOTCH/MYC signaling drives the aggressive SACC subtype (ACC-I) in a manner involving both MYC and MYC target genes^18^, in agreement with our findings. Moreover, these authors showed that MYC expression was higher in ACC-I than in ACC-II and was inversely correlated with prognosis. This evidence further supports that NICD1-MYB-MYC might be closely related to ACC-I subtypes. Our bioinformatic analysis unexpectedly identified MYC signaling as an alternative pathway for cancer cell proliferation upon the knockdown of both *MYB* and *NOTCH1* (Fig. 4h). Here, we have identified a function for MYB in MYC-driven metastatic SACC.

Additionally, our results demonstrated that NOTCH1 is related to the RA pathway (Fig. 5a, b, d). ATRA counteracts the NOTCH1–MYB–MYC axis by transcriptionally activating *NOTCH1* expression and repressing *MYB* expression (Fig. 6d), thereby suppressing lung metastasis in our nude mouse model. Furthermore, ATRA tended to increase lung metastatic nodule size in the *NOTCH1* knockdown metastasis model (Fig. 6e). These data suggest that NOTCH1 has dual functions in both cancer promotion and cancer suppression upon ATRA treatment. The tumor suppressor role of Notch signaling has been well established in many cancer types^51^, including head and neck squamous cell carcinoma (HNCC)^52^, hepatocellular carcinoma (HCC)^53^, and bladder carcinoma (BSCC)^54^. We identified the downregulation of RARA through RNA-seq and IHC data (Figs. 7d, 8b), implying that there is insufficient RA signaling in lung metastases. This finding warrants more attention for the future treatment of SACC.

## Supplementary information

Supplementary information

## Data Availability

All data supporting the findings of this study are available from the corresponding author upon reasonable request.
