# Supplementary material for: Increased retinoic acid signaling decreases lung metastasis in salivary adenoid cystic carcinoma by inhibiting the noncanonical Notch1 pathway
Source: Exp Mol Med. 2023 Mar 6;55(3):597–611. doi: 10.1038/s12276-023-00957-7 (PMC10073150; doi:10.1038/s12276-023-00957-7)
Supplement: Supplementary file 1 — Supplementary information [file 12276_2023_957_MOESM1_ESM.pdf]

Supplementary Fig 1

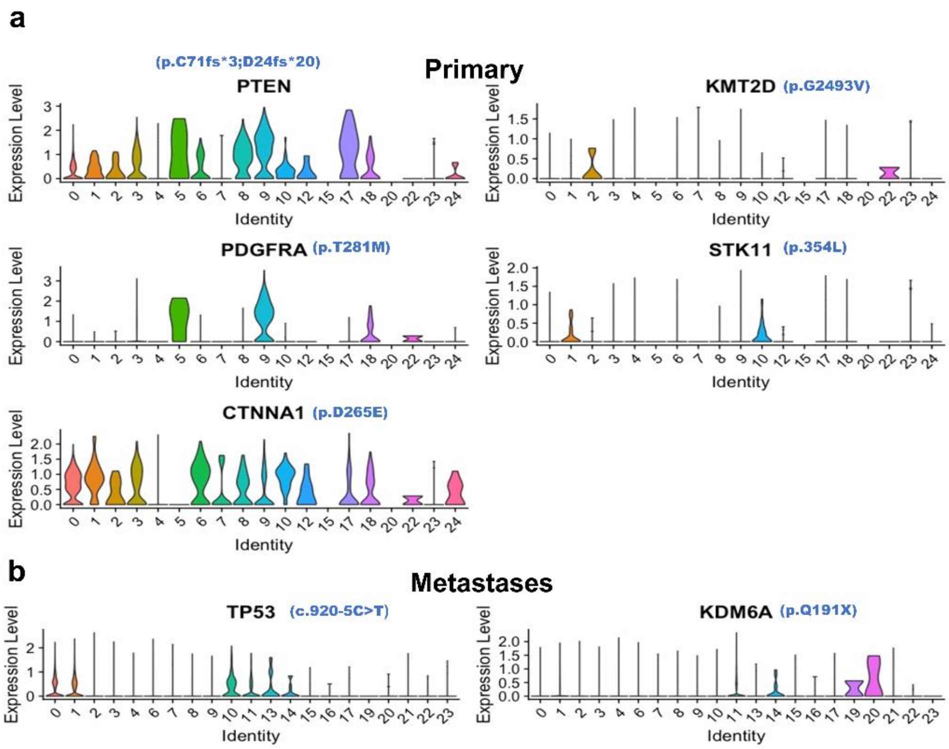

Supplementary Fig 2

a

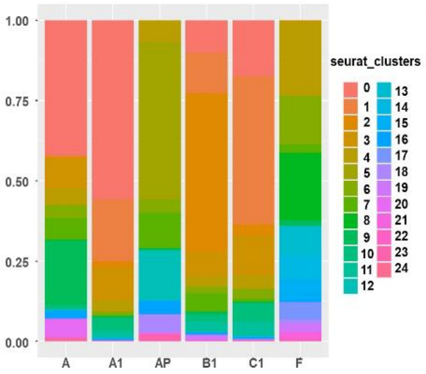

cluster	A	A1	AP	B1	C1	F
0	0.419	0.558	0.000	0.100	0.174	0.000
1	0.008	0.194	0.000	0.126	0.462	0.000
2	0.004	0.021	0.000	0.494	0.033	0.002
3	0.092	0.099	0.000	0.078	0.126	0.000
4	0.051	0.037	0.068	0.032	0.041	0.232
5	0.002	0.000	0.490	0.000	0.000	0.001
6	0.040	0.009	0.043	0.021	0.031	0.151
7	0.067	0.005	0.109	0.056	0.008	0.026
8	0.002	0.002	0.001	0.002	0.003	0.209
9	0.200	0.003	0.006	0.007	0.004	0.018
10	0.014	0.039	0.001	0.022	0.057	0.000
11	0.000	0.023	0.000	0.033	0.044	0.001
12	0.004	0.000	0.153	0.000	0.000	0.000
13	0.000	0.001	0.003	0.001	0.003	0.091
14	0.000	0.001	0.000	0.001	0.001	0.076
15	0.000	0.004	0.002	0.002	0.003	0.064
16	0.025	0.003	0.041	0.005	0.004	0.005
17	0.000	0.000	0.000	0.000	0.000	0.056
18	0.000	0.001	0.057	0.001	0.001	0.012
19	0.000	0.000	0.000	0.017	0.001	0.026
20	0.057	0.000	0.005	0.000	0.000	0.000
21	0.001	0.003	0.000	0.001	0.003	0.018
22	0.004	0.000	0.000	0.003	0.003	0.012
23	0.004	0.000	0.022	0.000	0.000	0.000
24	0.006	0.000	0.000	0.000	0.000	0.000

b

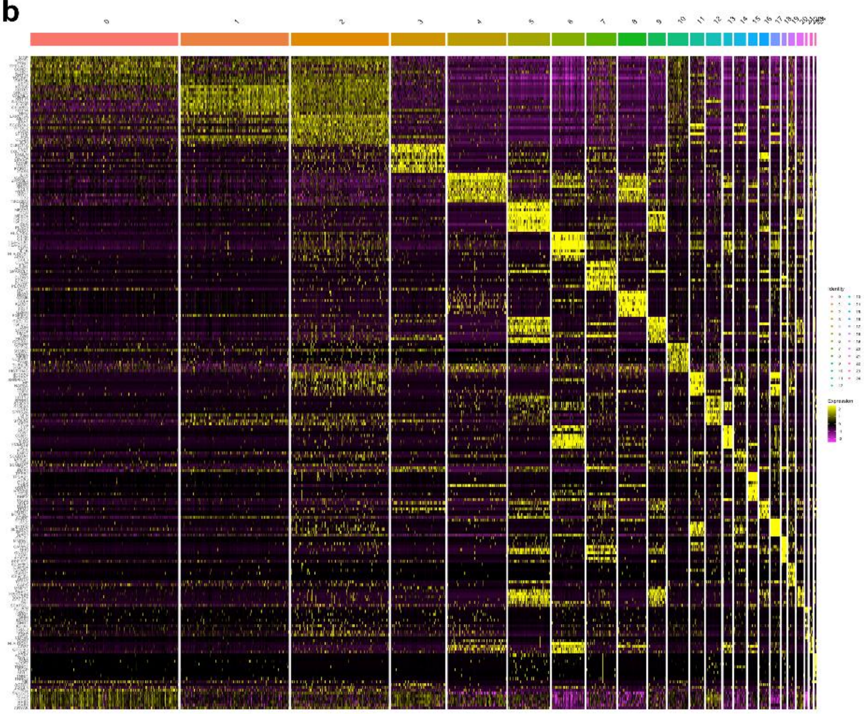

Supplementary Fig 3

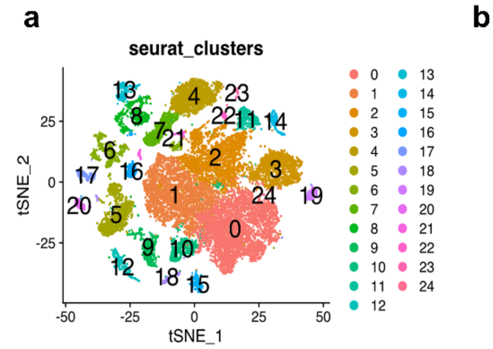

Cluster	Cell type	Canonical markers
0	Serous acinar cell	*EFHD1D, LTF, PRR4*
1	Mucous acinar cell	*BMP6, ALCAM, MUC5B*
2	Acinar cell	*BMP6, SCGB3A2,*
3	Myoepithelial cell	*TP63, MYL9*
4	CD8+ T cell	*CD3E, CD8A, PDCD1, CTLA4*
5	Fibroblast	*FN1, DCN, COL1A1, PDGFRA*
6	Vein endothelial cell	*ACKR1, VWF, PECAM1, ENG*
7	NK cell	*KLRF1, KLRD1*
8	Macrophage	*CD4, CD14, CD68, CD1C, CLEC10*
9	Fibroblast	*FN1; DCN; COL1A1, PDGFRA*
10	Unknown epithelial cell	*EPCAM, STMN1, MKI67, TOP2A*
11	Alveolar type II cell	*SFTPB, SFTPD, MUC1*
12	Basal cell	*KRT5, SFN*
13	Neutrophil	*S100A8, S100A9, IFITM2*
14	Alveolar type I cell	*AGER, CLIC5*
15	Mast cell	*MS4A2, CPA3, TPSAB1*
16	Dendritic cell	*FABP4, APOC1, C1QA*
17	Vascular Smooth Muscle	*ACTA2, MYL9, RGS5*
18	Fibroblast	*FN1, DCN, COL1A1, COL1A2*
19	Alveolar type II cell	*SFTPB, SFTPD, MUC1*
20	Lymphatic Endothelial cell	*PECAM1, PROX1*
21	Ciliated Cell	*FOXJ1, CCDC78*
22	Plasma Cell	*CD79A, CD27, SLAMF7*
23	B cell	*CD79A, MS4A1*
24	Undefined cell	

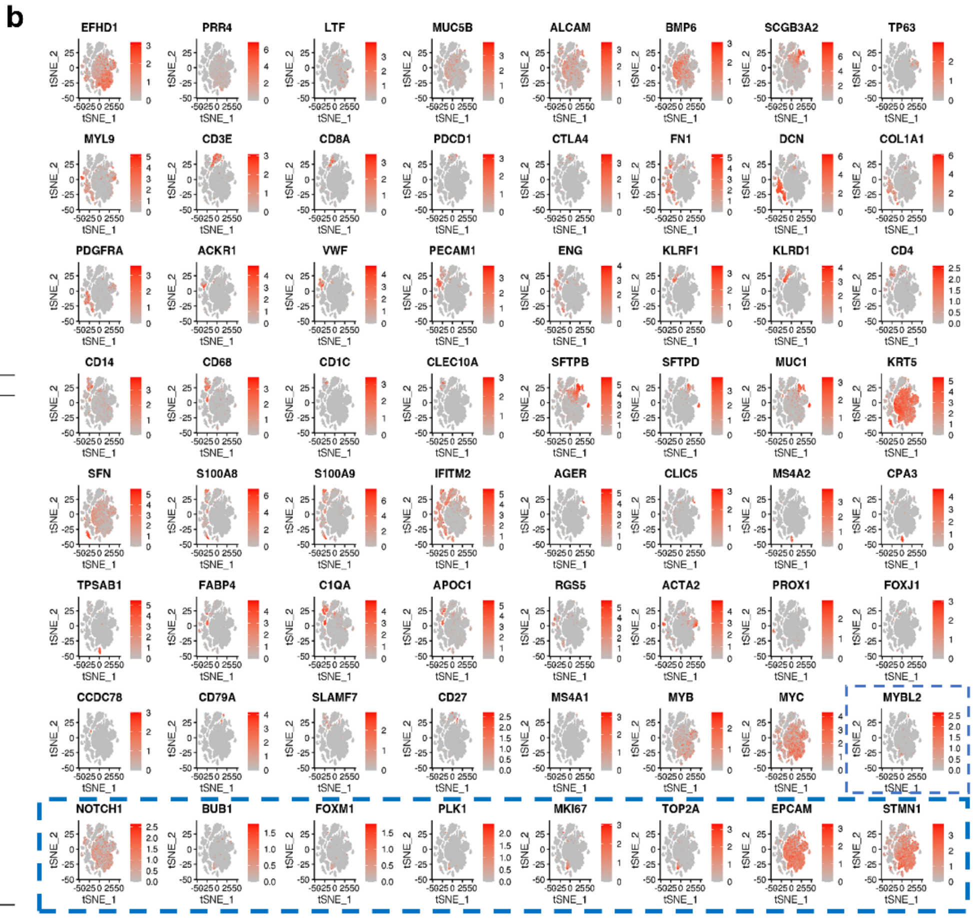

Supplementary Fig 4

a

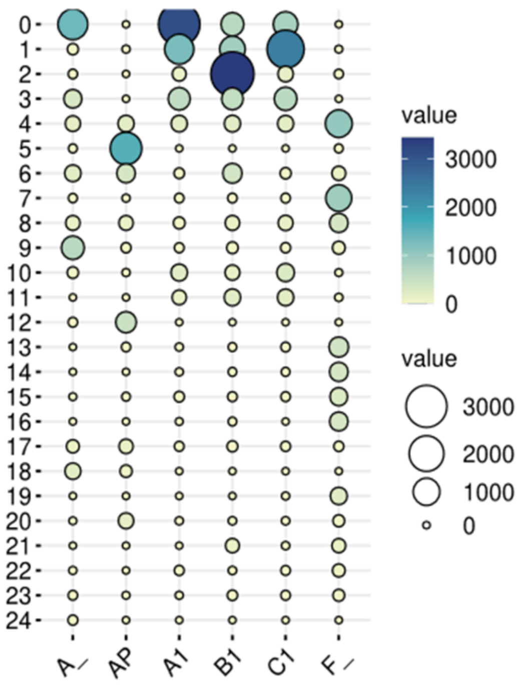

b

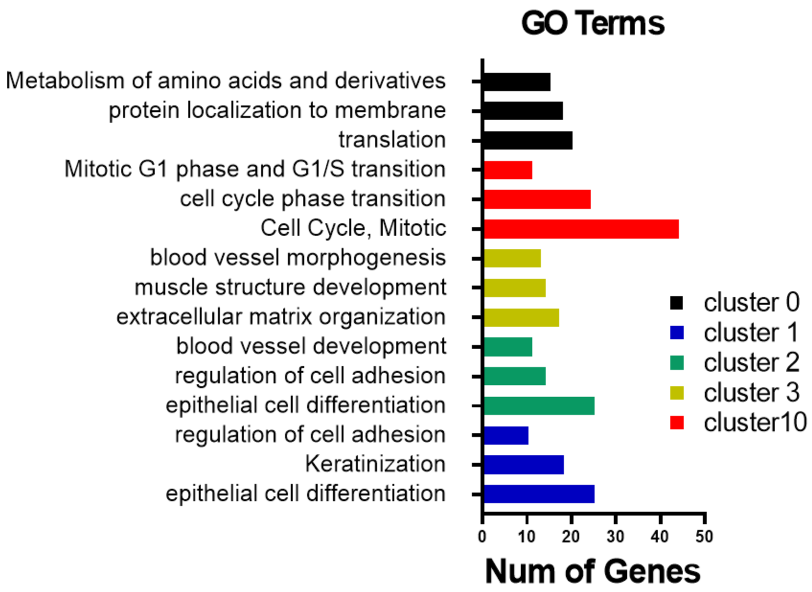

Supplementary Fig 5

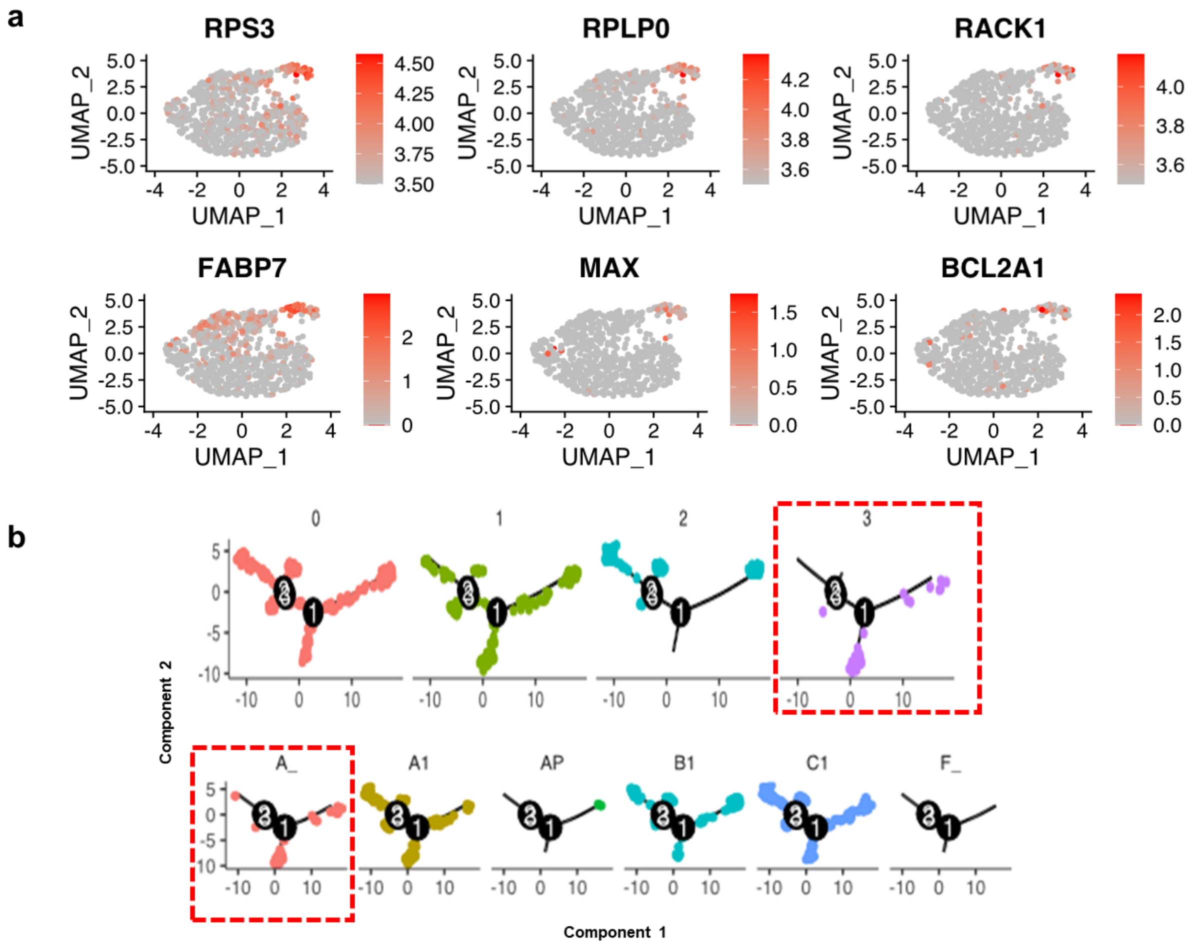

Supplementary Fig 6

a

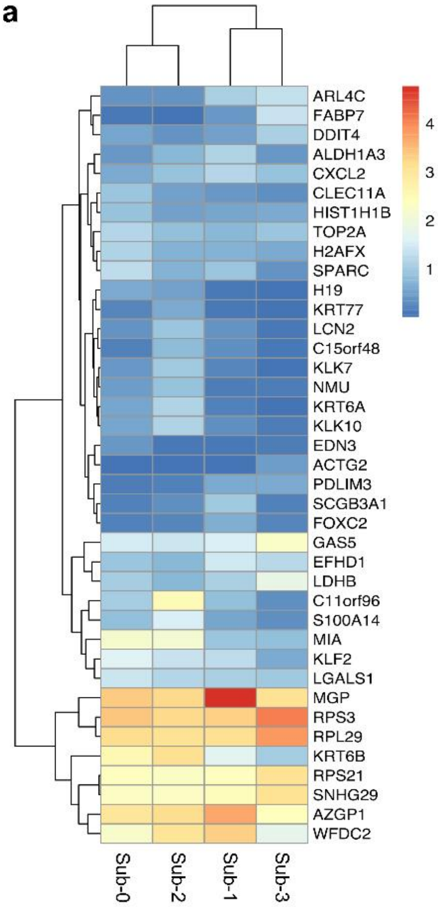

b

Top 20 signature genes

sub-0	sub-1	sub-2	sub-3
CLEC11A	SCGB3A1	C11orf96	FABP7
MIA	MGP	KRT6A	LDHB
SPARC	ARL4C	S100A14	ACTG2
TOP2A	WFDC2	NMU	GAS5
EDN3	ALDH1A3	KLK10	RPL29
KLF2	AZGP1	KLK7	ARL4C1
H2AFX	CXCL2	C15orf48	RPS3
H19	PDLIM3	KRT77	DDIT4
AL031668.2	EFHD1	LCN2	SNHG29
TUBB4B	FOXC2	KRT6B	RPS21
C6orf15	TPT1-AS1	MMP7	BCL2A1
PTTG1	SPARCL1	SERPINB2	TUBB2B
UBE2S	RGS10	SPON2	RPS19
CENPF	CCN1	S100A10	RPLP0
MKI67	CITED4	S100A6	RPS8
SLC44A1	SLC12A2	S100P	RACK1
HMGB2	OLFM4	CAMK2N1	VCAN
TPX2	PPP1R1A	MYBPC1	RPS3A
ARL6IP1	VIM	S100A16	RPSA
MSLN	HSPA1A	HSPB1	RPL36A

c

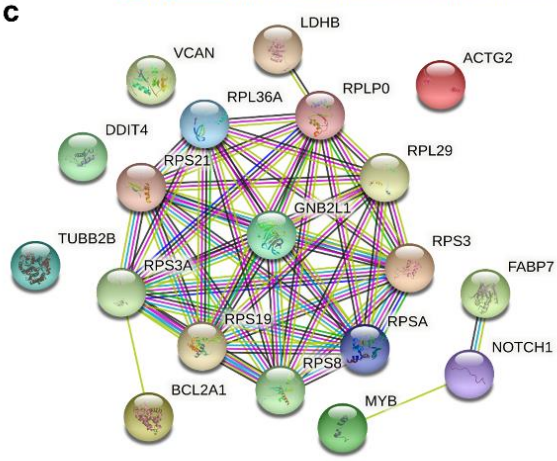

Supplementary Fig 7

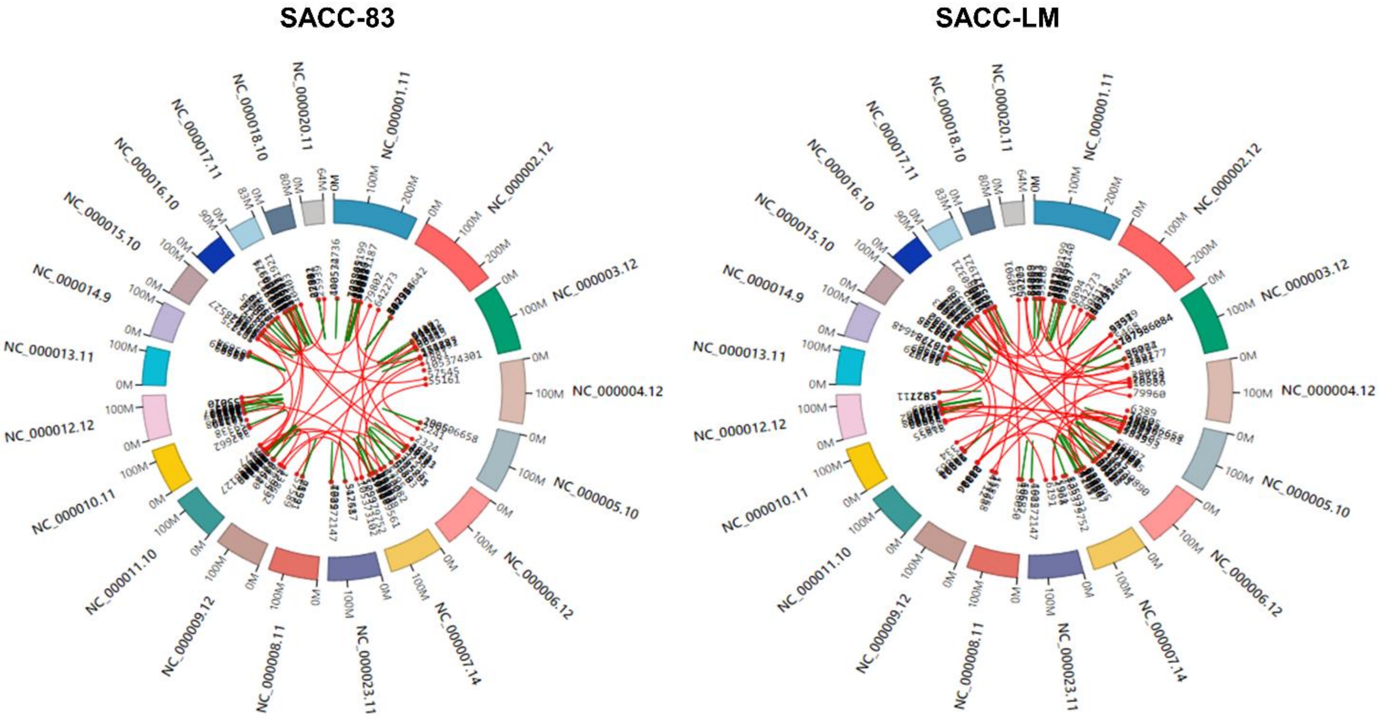

Supplementary Fig 8

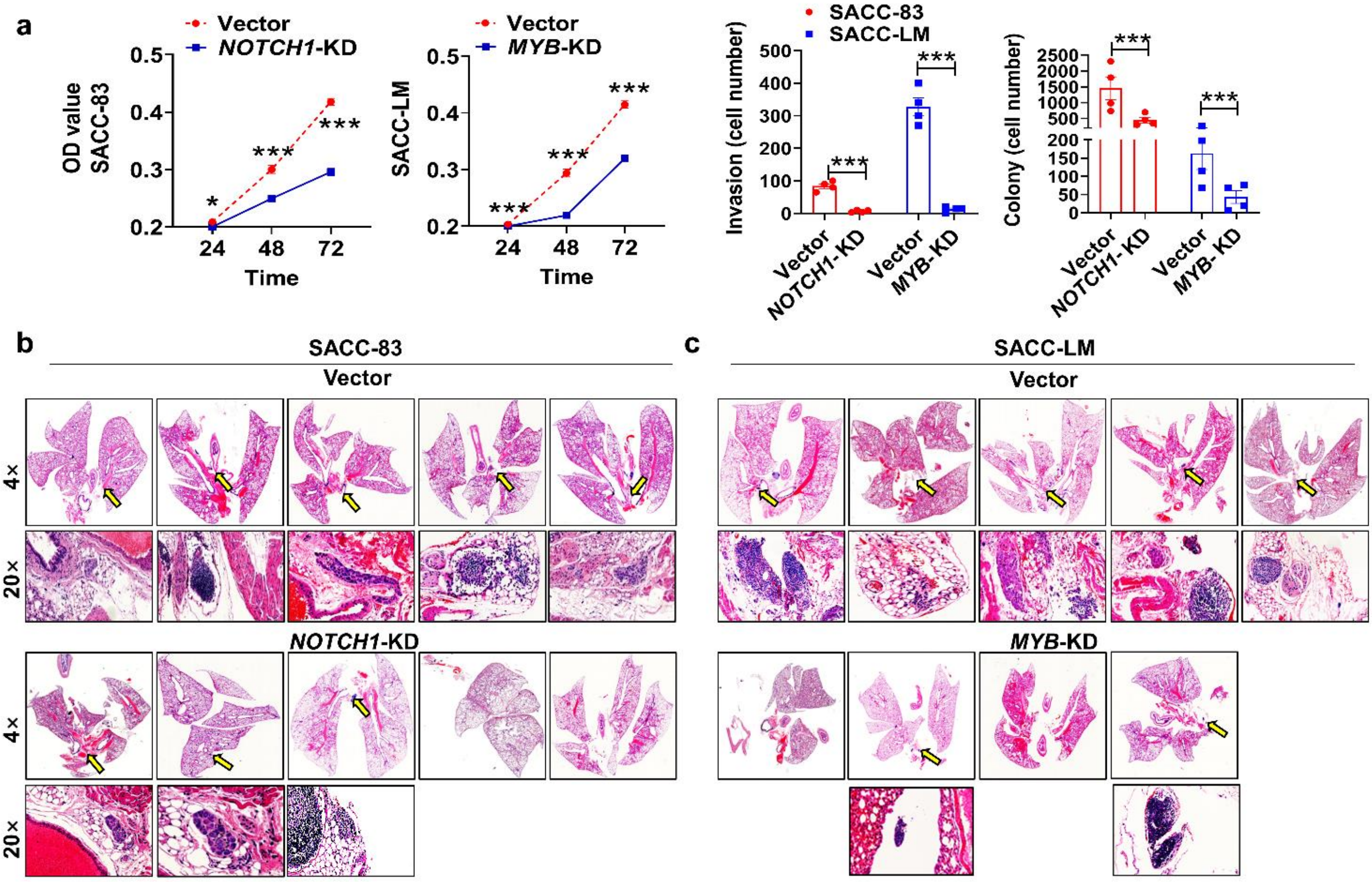

Supplementary Fig 9

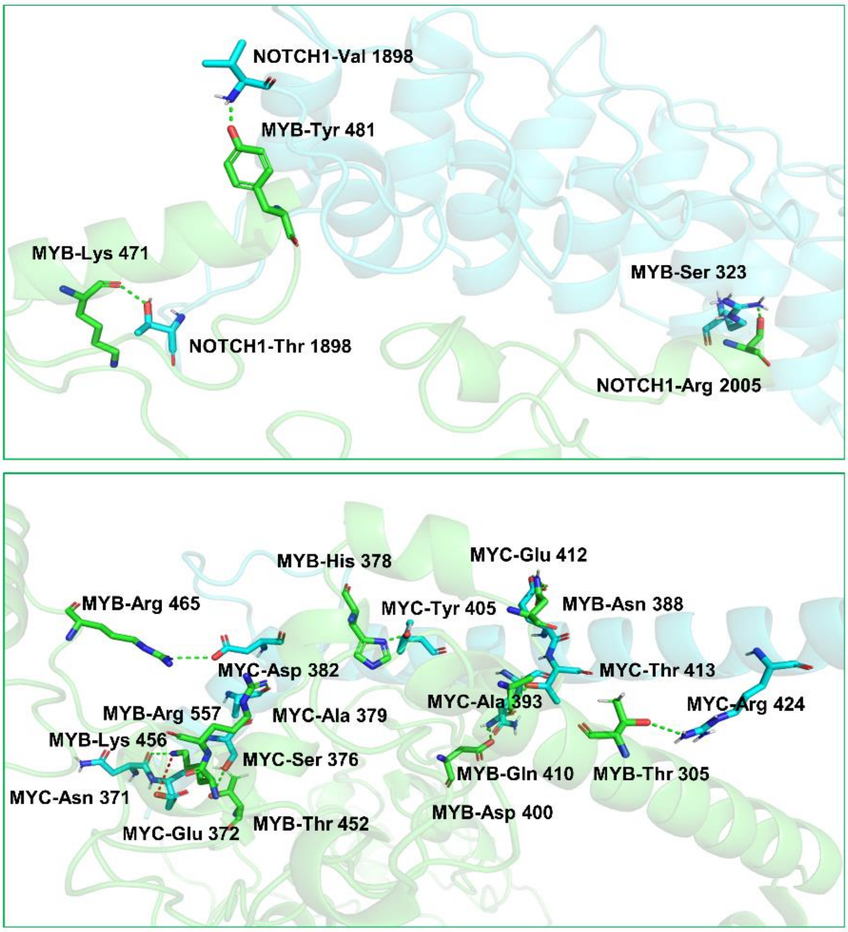

MYB Transcription activation domain：275-327

NICD1 domain：1754-2555

MYC bHLH domain : 354-406

MYC Leucine-zipper : 413-434

Green-hydrogen bond;

Red-salt bond

Supplementary tables

Supplementary Table 1 Clinical data and related experiments

Experiment type	Number of cases	Gender	Age	Tumour location	Exome target capture sequencing	Other
Single cell sequencing	1	Female	37	Tongue	a low tumour mutation burden (TMB)-1 mutation/Mb; PTEN (p.C71fs*3; D24fs*20), KMT2D (p.G2493V), PDGFRA (p.T281M), STK11 (p.354L), and CTNNA1 (p.D265E) mutations	The patient had an irregular mass at the tongue base, approximately 5.1×4.8 cm in size, with unclear margins and multiple cervical lymph node metastases
Single cell sequencing	1	Female	58	Lung metastases	TMB-0.4 mutations/Mb; KDM6A (p.Q191X) and TP53 (c.920-5C>T) mutations	The primary site was epiglottis ACC. Radiotherapy (60 GY) was applied after resection of the primary tumour, and surgical treatment was performed for pulmonary metastasis in the fifth year after surgery
ChIP-seq	1	Female	33	Maxillary sinus	MYB-NFIB gene fusion mutation	MYB-ChIP Seq
Immunohistochemistry	34	Male (16 cases), female (18 cases)	21-71 (Median age: 43.5)	Nasal sinus (13 cases), salivary gland (3 cases), larynx (2 cases), bronchus (2 cases), lacrimal gland (3 cases), external auditory canal (3 cases) and other parts (8 cases)		Detection of MYB, NICD1
Immunohistochemistry	37	Male (14 cases), female (23 cases)	25-69 (Median age: 53)	Nasal sinus (9 cases), lung metastasis (28cases)		Detection of MYB, NICD1, RARα, RARβ, RARγ, FABP7, NPM1, PRMT3

Supplementary Table 2 Cell type definition based on signature genes and their distribution ratio in tissues

Cluster	Cell type	Canonical markers	Literature	Cluster Ratio in primary tissue or lung metastases					
				**AP**	**A**	**F**	**A1**	**B1**	**C1**
0	Serous acinar cell	*EFHD1D, LTF, PRR4*	Maria OM et al. 2012; Plasschaert LW et al, 2018	0.000	0.419	0.000	0.558	0.100	0.174
1	Mucous acinar cell	*BMP6, ALCAM, MUC5B*	Maria OM et al. 2012	0.000	0.008	0.000	0.194	0.126	0.462
2	Acinar cell	*BMP6, SCGB3A2,*	Heikinheimo KA,1999	0.000	0.004	0.002	0.021	0.494	0.033
3	Myoepithelial cell	*TP63, MYL9*	Bilal et al. 2003	0.000	0.092	0.000	0.099	0.078	0.126
4	CD8+ T cell	*CD3E, CD8A, PDCD1, CTLA4*	Finkelstein et al. 1995; Banat et al. 2015 (k, i)	0.068	0.051	0.232	0.037	0.032	0.041
5	Fibroblast	*FN1, DCN, COL1A1, PDGFRA*	Crapo et al. 1982	0.490	0.002	0.001	0.000	0.000	0.000
6	Vein endothelial cell	*ACKR1, VWF, PECAM1, ENG*	Townsley et al. 2012	0.043	0.040	0.151	0.009	0.021	0.031
7	NK cell	*KLRF1, KLRD1*	Marquardt et al. 2017	0.109	0.067	0.026	0.005	0.056	0.008
8	Macrophage	*CD4, CD14, CD68, CD1C, CLEC10*	Crapo et al. 1982; Fehrenbach et al. 1994 (f)	0.001	0.002	0.209	0.002	0.002	0.003
9	Fibroblast	*FN1; DCN; COL1A1, PDGFRA*	Crapo et al. 1982	0.006	0.200	0.018	0.003	0.007	0.004
10	Unknown epithelial cell	*EPCAM, STMN1, MKI67, TOP2A*	Williams DW et al.2021	0.001	0.014	0.000	0.039	0.022	0.057
11	Alveolar type II cell	*SFTPB, SFTPD, MUC1*	Crapo et al. 1982; Fehrenbach et al. 1994	0.000	0.000	0.001	0.023	0.033	0.044
12	Basal cell	*KRT5, SFN*	Boers et al. 1998	0.153	0.004	0.000	0.000	0.000	0.000
13	Neutrophil	*S100A8, S100A9, IFITM2*	Finkelstein et al. 1995; Banat et al. 2015	0.003	0.000	0.091	0.001	0.001	0.003
14	Alveolar type I cell	*AGER, CLIC5*	Crapo et al. 1982 (f)	0.000	0.000	0.076	0.001	0.001	0.001
15	Mast cell	*MS4A2, CPA3, TPSAB1*	Finkelstein et al. 1995; Banat et al. 2015	0.002	0.000	0.064	0.004	0.002	0.003
16	Dendritic cell	*FABP4, APOC1, C1QA*	Townsley et al. 2012	0.041	0.025	0.005	0.003	0.005	0.004
17	Vascular smooth muscle cell	*ACTA2, MYL9, RGS5*	Crapo et al. 1982; Fehrenbach et al. 1994	0.000	0.000	0.056	0.000	0.000	0.000

18	Fibroblast	*FN1, DCN, COL1A1, COL1A2*	Crapo et al. 1982	0.057	0.000	0.012	0.001	0.001	0.001
19	Alveolar type II cell	*SFTPB, SFTPD, MUC1*	Crapo et al. 1982; Fehrenbach et al. 1994 (f)	0.000	0.000	0.026	0.000	0.017	0.001
20	Lymphatic endothelial cell	*PECAM1, PROX1*	Kambouchner et al. 2009; Sozio et al. 2012	0.005	0.057	0.000	0.000	0.000	0.000
21	Ciliated epithelial cell	*FOXJ1, CCDC78*	Crapo et al. 1982 (f)	0.000	0.001	0.018	0.003	0.001	0.003
22	Plasma cell	*CD79A, CD27, SLAMF7*	Banat et al. 2015 (k)	0.000	0.004	0.012	0.000	0.003	0.003
23	B cell	*CD79A, MS4A1*	Finkelstein et al. 1995; Banat et al. 2015 (k)	0.022	0.004	0.000	0.000	0.000	0.000
24	Undefined cell			0.000	0.006	0.000	0.000	0.000	0.000

Supplementary Table 3 MYB-ChIP data region upstream of 2 kb in a patient with MYB-NFIB fusion

chr	peak start	peak end	gene id	symbol
chr1	2627219	2627313	100287898	TTC34
chr1	29567178	29567357	10076	PTPRU
chr1	43094751	43094904	728621	CCDC30
chr1	56996120	56996262	8613	PPAP2B
chr1	63733146	63733265	199899	LINC00466
chr1	93197142	93197256	7813	EVI5
chr1	145277189	145277332	100288142	LOC100288142
chr1	145277189	145277332	388677	NOTCH2NL
chr1	145277189	145277332	400818, (100132406)	
chr1	145352395	145352464	100132406	NBPF10
chr1	145352395	145352464	100288142	LOC100288142
chr1	145352395	145352464	101929780	LOC101929780
chr1	145352395	145352464	400818, (100132406)	
chr1	184799385	184799534	116496	FAM129A
chr1	208412343	208412488	5362	PLXNA2
chr1	225775101	225775179	55740	ENAH
chr10	24762815	24762981	56243	KIAA1217
chr10	74454859	74455034	90550	MCU
chr10	90145709	90145965	55328	RNLS
chr10	104104110	104104261	8729	GBF1
chr10	104536760	104536935	54838	WBP1L
chr10	104756955	104757116	54805	CNNM2
chr11	19996677	19996832	89797	NAV2
chr11	20489389	20489540	10196	PRMT3
chr11	44146959	44147102	2132	EXT2
chr11	84539600	84539746	1740	DLG2
chr11	93885455	93885602	24145	PANX1
chr11	106705859	106706023	2977	GUCY1A2
chr11	126489666	126489779	84623	KIRREL3
chr11	133797470	133797607	22997	IGSF9B
chr12	21597996	21598136	79912	PYROXD1
chr13	26540878	26541017	51761	ATP8A2
chr13	35187301	35187448	100874179	LINC00457
chr13	41638436	41638567	11193	WBP4
chr13	78314454	78314597	122060	SLAIN1
chr13	113389755	113389902	23250	ATP11A
chr14	23895403	23895536	4625	MYH7
chr14	76242716	76242832	23093	TTLL5
chr14	89673045	89673161	1112	FOXN3
chr14	94689072	94689192	57718	PPP4R4
chr15	22568624	22568790	646396	REREP3
chr15	35835687	35835831	89978	DPH6
chr15	42247028	42247169	30844	EHD4

chr15	57183340	57183510	145783	LOC145783
chr15	58947500	58947648	102	ADAM10
chr15	61148258	61148395	6095	RORA
chr15	80742868	80743013	9915	ARNT2
chr15	89846643	89846764	55215	FANCI
chr16	20817823	20818082	81691	LOC81691
chr16	58556466	58556590	23019	CNOT1
chr16	77379580	77379722	170692	ADAMTS18
chr16	84091117	84091241	8720	MBTPS1
chr17	3496656	3496793	7442	TRPV1
chr17	39093738	39093859	25984	KRT23
chr17	61556437	61556587	1636	ACE
chr17	62559818	62559958	64750	SMURF2
chr18	4271874	4272014	284215	DLGAP1-AS5
chr18	4271874	4272014	9229	DLGAP1
chr18	5238788	5238913	339290	LINC00667
chr18	21362008	21362165	3909	LAMA3
chr19	8371212	8371412	51293	CD320
chr19	9213824	9213943	390882	OR7G2
chr19	12035886	12036010	90592	ZNF700
chr19	36505910	36506057	25999	CLIP3
chr19	46143941	46144083	24139	EML2
chr19	50811091	50811231	79784	MYH14
chr19	52478105	52478242	101669766	HCCAT3
chr19	52478105	52478242	59348	ZNF350
chr2	21912650	21912794	645949	LOC645949
chr2	26562518	26562709	165082	GPR113
chr2	56487010	56487151	114800	CCDC85A
chr2	74294044	74294178	200424	TET3
chr2	122179571	122179675	23332	CLASP1
chr2	131126053	131126201	26469	PTPN18
chr2	168091132	168091278	129446	XIRP2
chr2	173305237	173305367	3655	ITGA6
chr2	174027791	174027932	51776	ZAK
chr2	212402148	212402282	2066	ERBB4
chr2	231305936	231306079	6672	SP100
chr2	234294309	234294452	8527	DGKD
chr20	13699401	13699543	51575	ESF1
chr20	33323918	33324064	23054	NCOA6
chr21	11049910	11050196	85316	BAGE5
chr21	11049910	11050196	85317	BAGE4
chr21	11049910	11050196	85318	BAGE3
chr21	11049910	11050196	85319	BAGE2
chr21	34813911	34814057	757	TMEM50B
chr21	41426412	41426522	1826	DSCAM
chr21	42581288	42581436	25825	BACE2

chr22	36725581	36725742	4627	MYH9
chr22	40994555	40994695	57591	MKL1
chr3	3201409	3201543	51185	CRBN
chr3	4467685	4467820	285362	SUMF1
chr3	10260367	10260496	3656	IRAK2
chr3	10541852	10542003	491	ATP2B2
chr3	15711443	15711592	23243	ANKRD28
chr3	71098319	71098500	27086	FOXP1
chr3	73560874	73561122	23024	PDZRN3
chr3	85843181	85843325	253559	CADM2
chr3	118642146	118642279	152404	IGSF11
chr3	124259811	124259952	8997	KALRN
chr3	177163367	177163517	100505566	LINC00578
chr3	194943488	194943642	152002	XXYLT1
chr3	196946844	196946967	1739	DLG1
chr4	41122956	41123156	323	APBB2
chr4	165568813	165568956	100847071	MIR5684
chr5	65012331	65012480	54557	SGTB
chr5	76579784	76579950	8622	PDE8B
chr5	83536371	83536508	10085	EDIL3
chr5	90407373	90407612	84059	GPR98
chr5	100163542	100163692	7903	ST8SIA4
chr5	138390135	138390278	64374	SIL1
chr6	3106742	3106873	8737	RIPK1
chr6	20842174	20842365	54901	CDKAL1
chr6	69354077	69354206	577	BAI3
chr6	71548654	71548811	60682	SMAP1
chr6	75953350	75953585	1347	COX7A2
chr6	109242827	109243096	101929716	ARMC2-AS1
chr6	109242827	109243096	84071	ARMC2
chr6	136979849	136979988	4217	MAP3K5
chr6	151197823	151197968	25902	MTHFD1L
chr7	14309220	14309385	1607	DGKB
chr7	87837953	87838083	6717	SRI
chr7	90820544	90820684	5218	CDK14
chr7	92268945	92269293	1021	CDK6
chr7	116411298	116411444	4233	MET
chr7	144478675	144478820	27010	TPK1
chr7	151999562	151999684	58508	KMT2C
chr8	8743560	8743703	9258	MFHAS1
chr8	17173353	17173494	9108	MTMR7
chr8	22201404	22201546	55124	PIWIL2
chr8	39539842	39539979	8749	ADAM18
chr8	71556906	71557027	286190	LOC286190
chr8	71556906	71557027	51110	LACTB2
chr8	103420585	103420736	51366	UBR5

chr9	2179762	2179886	6595	SMARCA2
chr9	9455397	9455531	5789	PTPRD
chr9	87326945	87327140	4915	NTRK2
chr9	138848056	138848201	10422	UBAC1
chrUn_gl000220	117825	125419	100507412	LOC100507412
chrUn_gl000220	117825	125419	100861532	RNA45S5
chrX	68909990	68910119	1896	EDA
chrX	84558578	84558730	79983	POF1B
chrX	101870495	101870642	100528062	ARMCX5-GPRASP2
chrX	111274449	111274574	7224	TRPC5
chrX	117666245	117666377	139818	DOCK11
chrX	118117459	118117599	79836	LONRF3
chrX	123026386	123026518	331	XIAP
chrX	123614903	123615040	10178	TENM1
chrX	138721846	138721996	4168	MCF2
chrX	153227431	153227576	3054	HCFC1

Supplementary Table 4 Summary of materials used in the experiments

Experiment	Antibodies	Source	Concentration	Identifier
Immunohistochemical	anti-c-Myb	Abcam, Cat# ab45150	1:100	AB_778878
	anti-cleaved Notch1	Cell Signaling Technology, Cat# 4147	1:80	AB_2153348
	anti-RARα	Proteintech, Cat# 10331-1-AP	1:100	AB_2177742
	anti-RARβ	Abcam, Cat# ab124701	1:100	AB_10975008
	anti-RARγ	Cell Signaling Technology, Cat# 8965	1:100	AB_10998934
	anti-FABP7	Proteintech, Cat# 14836-1-AP	1:100	AB_2100458
	anti-PRMT3	Abcam, Cat# ab191562	1:100	NA
	anti-NPM	Cell Signaling Technology, Cat# 3542	1:100	AB_2155178
Western blot	anti-GAPDH	Proteintech, Cat# 60004-1-Ig	1:5000	AB_2107436
	anti-β-Actin	Abcam, Cat# AC026	1:5000	AB_2768234
	anti-c-Myb	Cell Signaling Technology, Cat# 12319	1:1000	AB_2716637
	anti-cleaved Notch1	Cell Signaling Technology, Cat# 4147	1:1000	AB_2153348
	anti-c-MYC	Proteintech, Cat# 10828-1-AP	1:1000	AB_2148585
	anti-MAZ	Proteintech, Cat#21068-1-AP	1:1000	AB_2878805
	anti-HES1	Cell Signaling Technology, Cat#11988	1:1000	AB_2728766
	anti-RARα	Proteintech, Cat# 10331-1-AP	1:1000	AB_2177742
	anti-RARβ	Abcam, Cat# ab124701	1:1000	AB_10975008
	anti-RARγ	Cell Signaling Technology, Cat# 8965	1:1000	AB_10998934
Co-immunoprecipitation	anti-cleaved Notch1	Cell Signaling Technology, Cat# 2421	1:1000	AB_2314204
	anti-c-Myb	Cell Signaling Technology, Cat# 12319	1:1000	AB_2716637

	anti-c-MYC	Proteintech, Cat# 10828-1-AP	1:200	AB_2148585
	Normal Rabbit IgG	Cell Signaling Technology Cat#2729S	1:200	AB_1031062
Chemicals	ATRA	MedChemExpress, HY-14649	Cell:1 μM; Animal:5-10mg/kg	
	DAPT	MedChemExpress, HY-13027	Cell: 20 μM; Animal:10mg/kg	
	GW9662	MedChemExpress, HY-16578	1 μM	
	AGN193109	MedChemExpress, HY-u00449	1 μM	
Instruments	PV two-step IHC kit	ZSGB-BIO, PV-9000		
	Leica light microscope	Germany, DM6		
	Lipofectamine 8000TM	Beyotime, C0533		
	2100 Bioanalyzer	Agilent		
	In vivo image system	PerkinElmer, Lumina II		
	CCK8 kit	Applygen, E1008-200		
	24-well cell culture inserts	Corning,3422		
	RNA extraction kit	Yishan, ES-RN001		
	Reverse transcription kit	Takara, RR037A		
Other	Protein A/G Agarose	Sigma, P5641		
	T4 DNA Ligase	Thermo, EL0011		
	penicillin–streptomycin solution	Biosharp, BL505A		
	pCDHO-neo-MYB-3-Flag	Vigene, CH898248		
	pcDNA3.1-*MYC*-HA plasmid	ChemicalBook		
	T4 ligase	Thermo, EL0011		
	E. coli	Thermo, FD0274		
	293T cells	CCLV, Cat# CCLV-RIE 1018		

Supplementary Table 5 sh primers used in knockdown experiments

Name	sh Primer Sequence
MYB	shRNA-*MYB*-1-F：5'-CCGGAACAGAATGGAACAGATGACCTCGAGGTCATCTGTTCCATTCTGTTCTTTTT-3'
	shRNA-MYB-1-R：5'-AATTAAAAAAACAGAATGGAACAGATGACCTCGAGGTCATCTGTTCCATTCTGTTC-3'
	shRNA-*MYB*-2-F：5'-CCGGCCAGATTGTAAATGCTCATTTCTCGAGAAATGAGCATTTACAATCTGGTTTTT-3'
	shRNA-*MYB*-2-R：5'-AATTAAAAACCAGATTGTAAATGCTCATTTCTCGAGAAATGAGCATTTACAATCTGG-3'
NOTCH1	shRNA-NOTCH1-1-F: 5'-CCGGAGGTCAGTGTGGAGGTGGATTCTCGAGAATCCACCTCCACACTGACCTTTTTTT-3'
	shRNA-*NOTCH1*-1-R: 5'-AATTAAAAAAGGTCAGTGTGGAGGTGGATTCTCGAGAATCCACCTCCACACTGACCTT-3'
	shRNA-*NOTCH1*-2-F: 5'-CCGGGCGCTGATCAGCGGTATTGAACTCGAGTTCAATACCGCTGATCAGCGCTTTTTT-3'
	shRNA-*NOCTH1*-2-R: 5'-AATTAAAAAGCGCTGATCAGCGGTATTGAACTCGAGTTCAATACCGCTGATCAGCGCT-3'
MYC	shRNA-*MYC*-1-F：5'-CCGGCCTGAGACAGATCAGCAACAACTCGAGTTGTTGCTGATCTGTCTCAGGTTTTT-3'
	shRNA-*MYC*-1-R：5'-AATTAAAAACCTGAGACAGATCAGCAACAACTCGAGTTGTTGCTGATCTGTCTCAGG-3'
	shRNA-*MYC*-2-F：5'-CCGGGCTTCACCAACAGGAACTATGCTCGAGCATAGTTCCTGTTGGTGAAGCTTTTT-3
	shRNA-*MYC*-2-R：5'-AATTAAAAAGCTTCACCAACAGGAACTATGCTCGAGGTCATCTGTTCCATTCTGTTC-3'

Supplementary Table 6 Primers for qPCR

Name	Primer Sequence
MYB	F: 5’- GGCGAGCCCCTTGCA-3’<br>R: 5’-CTCCTCCATCTTTCCACAGGAT-3’
NOTCH1	F: 5′-GGTGAGACCTGCCTGAATG-3′<br>R: 5′-GTTGGGGTCCTGGCATC-3′
MYC	F: 5’-TGCTCCATGAGGAGACACC-3′<br>R: 5’-CTCTGACCTTTTGCCAGGAG-3′
HES1	F: 5’-AGGCGGACATTCTGGAAATG-3′<br>R: 5’-CGGTACTTCCCCAGCACACTT-3′
MYBL2	F: 5’- GATGTCCACACTGCCCAAGT-3’<br>R: 5’-CAGGTGTCGTGAAGTGGCTT-3
MAZ	F: 5’- GGATCACCTCAACAGTCACGTC-3’<br>R: 5’-GGCACTTTCTCCTCGTGTCGTA-3’
GAPDH	F: 5'- GACAACTTTGGCATCGTGGA-3'<br>R: 5'-ATGCAGGGATGATGTTCTGG-3'

Supplementary figure legends

Supplementary Fig 1. Mutation types and violin plots of mutated genes. Violin plots depicting the expression levels of the mutated genes in the 25 clusters, including *PTEN* (p.C71fs*3; D24fs*20), *KMT2D* (p.G2493V), *PDGFRA* (p.T281M), *STK11* (p.354L), and *CTNNA1* (p.D265E) mutations in primary samples (a) and *KDM6A* (p. Q191X) and *TP53* (c.920-5C>T) mutations in lung metastases (b). Some mutated genes showed aberrant expression in specific cancer cell clusters.

Supplementary Fig 2. Distribution of different clusters in six samples and their uniquely enriched genes. (a) Frequency distribution plots and their values in six samples. (b) Heatmap of DEGs showing enriched genes for each cell cluster.

Supplementary Fig 3. Definition of cell types based on unique genes. (a) The definitions of the cell types are shown by a t-SNE plot and known marker genes. (b) t-SNE plots showing the expression of known marker genes of each cell cluster. The blue dashed boxes highlight the unique genes and known stem cell genes in cluster 10.

Supplementary Fig 4. Analysis of the number and GO function of cluster 10. (a) Bubble graph showing the number of cell clusters (size scale) and the z-scores (colour scale) for the DEGs. (b) GO analysis of 4 cell subtypes after sub-clustering of cluster 10.

Supplementary Fig 5. Analysis of the unique genes and trajectories of

subcluster 10. (a) t-SNE plots showing the expression of unique marker genes of subtype 3 from cluster 10. (b) Pseudotime trajectory analysis of all subtype cells from cluster 10 and all samples by Monocle 2. Trajectories of subtype 3 and the primary tumour (A) are highlighted in red dashed boxes.

Supplementary Fig 6. Identification of differentially expressed genes and the interaction network for cell subtypes in cluster 10. (a) Heatmap showing the top 50 DEGs in the four subtypes. (b) Top 20 DEGs. (c) The PPI network established the interactions between 20 DEGs and *MYB* or *NOTCH1*.

Supplementary Fig 7. Circos plots of fusions in SACC-83 cells and SACC-LM cells.

Supplementary Fig 8. Both NOTCH1 and MYB promote SACC cell growth and lung metastasis. (a) Statistical analysis of cell proliferation, invasion, and colony formation at different time points after *NOTCH1* knockdown in SACC-83 cells or MYB knockdown in SACC-LM cells. The *p* values were calculated using the paired two-tailed Student's t-test. Triple repeat data are presented as the mean±SEM. $p<0.05$ (*), $p<0.01$ (**), $p<0.001$ (***). (b) HE images showing lung metastases induced by the left intraventricular injection of SACC-83-*NOTCH1*-KD, SACC-LM-*MYB*-KD, or control cells at 28 days (4-5 per group). The number of metastases was calculated and compared between groups (Fig. 3f).

Supplementary Fig 9. The reciprocal interactions between NICD1 and MYB or between MYC and MYB predicted with AlphaFold. Crystal structures of NICD1 (1754-2555) and MYC (350-439) were obtained from NCBI

Structure (PDB ID: 6PY8; PDB ID: 5I4Z). The crystal structures of MYB (200-500) and MYC (100-300) were predicted with AlphaFold2. Interactive docking of protein–protein complexes was performed in the ZDOCK server. Images were exported from PyMOL 2.5.
